# Associations of diet quality indices with all-cause and cause-specific mortality among Japanese adults in the Takayama study

**DOI:** 10.1017/S0007114525106077

**Published:** 2026-04-14

**Authors:** Fumi Oono, Keiko Wada, Michiyo Yamakawa, Masaaki Sugino, Tomoka Mori, Shino Oba, Kentaro Murakami, Chisato Nagata

**Affiliations:** 1 Department of Epidemiology and Preventive Medicine, Graduate School of Medicine, Gifu University, Gifu, Japan; 2 Department of Social and Preventive Epidemiology, Division of Health Sciences and Nursing, Graduate School of Medicine, The University of Tokyo, Tokyo, Japan; 3 Graduate School of Health Sciences, Gunma University, Maebashi, Gunma, Japan; 4 Department of Social and Preventive Epidemiology, School of Public Health, The University of Tokyo, Tokyo, Japan

**Keywords:** Quality of diet, Dietary quality, Dietary patterns, Death, Prospective study, Japanese

## Abstract

This prospective study investigated the associations of various diet quality indices with mortality in Japan. Participants were 13 355 men and 15 724 women from the Takayama study. Eight diet quality indices were assessed using an FFQ: the Dietary Approaches to Stop Hypertension, alternative Mediterranean diet scores, Healthy Eating Index-2015, Alternate Healthy Eating Index-2010, Nutrient Rich Food Index 9.3, Diet Quality Score for Japanese, Japanese Food Guide Spinning Top and twelve-item Japanese Diet Index. Cox proportional models estimated hazard ratios and 95 % CI for all-cause and cause-specific mortality in a 1 sd difference for each index, with adjustment for confounders. During a mean follow-up of 14·1 years, 5339 deaths were recorded. Hazard ratios (95 % CI) per 1 sd higher index were 0·90 (0·87, 0·93) for Alternate Healthy Eating Index-2010, 0·92 (0·89, 0·95) for Diet Quality Score for Japanese, 0·93 (0·91, 0·96) for Nutrient Rich Food Index 9.3, 0·94 (0·92, 0·97) for alternative Mediterranean diet and Dietary Approaches to Stop Hypertension, 0·94 (0·91, 0·97) for Japanese Food Guide Spinning Top, 0·94 (0·91, 0·98) for twelve-item Japanese Diet Index and 0·97 (0·94, 0·996) for Healthy Eating Index-2015. Similar protective associations were observed for CVD mortality, but not for cancer mortality. These findings suggest that all eight indices are associated with lower mortality and that the strength of associations varies across indices; the Alternate Healthy Eating Index-2010 showed relatively strong associations, followed by the Diet Quality Score for Japanese, whereas the associations of the Healthy Eating Index-2015 appeared relatively weaker in this Japanese population.

A suboptimal diet is a major modifiable risk factor contributing to global deaths^(1)^. Overall dietary intake is often evaluated using diet quality indices, which are summary scores of dietary intake based on previous knowledge or hypotheses concerning the health effects of dietary components^(2)^. For example, the Healthy Eating Index was developed based on dietary guidelines in the USA^(3)^ and was then, followed by the development of the Alternate Healthy Eating Index by considering evidence on diet and chronic disease^(4)^. To date, various diet quality indices have been developed, and their inverse associations with mortality have been reported^(5–21)^. In Japan, where dietary habits have attracted global interest due to the longevity of the Japanese^(22)^, two types of index have been associated with lower mortality: indices assessing adherence to the Japanese diet^(9–12)^ and the Japanese Food Guide Spinning Top (JFGST), which assesses adherence to national dish- and food-based dietary guidelines in Japan^(13,14)^.

These Japanese indices, however, did not necessarily reflect evidence on diet-health associations in their development process. For example, the Japanese Diet Index, including the twelve-item Japanese Diet Index (JDI12), assesses adherence to traditional dietary patterns in Japan, including higher intake of rice (mainly refined rice) and lower intake of coffee^(23,24)^, which may contradict the associations of these with health outcomes, including mortality^(25,26)^. Similarly, the JFGST categorises foods with possibly different health effects as belonging to the same category (e.g., whole grains or refined grains as a staple dish and fish or processed meat as a fish and meat dish)^(3,4,25)^. Accordingly, diet quality indices that are consistent with evidence on foods and health outcomes may show stronger associations than those examined. Recently, the evidence-based index Diet Quality Score for Japanese (DQSJ) was developed by integrating existing diet quality indices from Western countries with the characteristics and health impact of dietary intake in Japan^(27)^. A previous study suggested that the DQSJ has stronger associations with cardiometabolic status than the JFGST^(28)^, but its associations with other health outcomes have not yet been examined.

In addition, considering the similarity of dietary concerns in Japan and worldwide^(27)^, diet quality indices that showed consistent associations with lower mortality worldwide may also be beneficial in Japan. These include the Healthy Eating Index, Alternate Healthy Eating Index, Mediterranean diet scores and Dietary Approaches to Stop Hypertension (DASH) scores^(5–8)^. To our knowledge, however, no Japanese study has examined associations of these indices with all-cause mortality. Additionally, while nutrient-based indices, such as the Nutrient Rich Food Index 9.3 (NRF9.3)^(29,30)^, may have the advantage of being independent of specific food groups, making them applicable to various populations^(2,31)^, their associations with mortality have been little examined^(15–17)^, particularly in non-Western countries^(17)^. It remains largely unknown which diet quality indices are more effective for predicting mortality in individual countries^(18–21)^, especially in Japan.

Here, as part of efforts to explore diet quality indices for predicting health outcomes in Japan, we examined associations of various diet quality indices, including DASH, alternate Mediterranean diet (AMED) score, Healthy Eating Index-2015 (HEI-2015), Alternate Healthy Eating Index-2010 (AHEI-2010), NRF9.3, DQSJ, JFGST and JDI12, with all-cause and cause-specific mortality in the Japanese population. We sought to present comparable estimates of associations with mortality for these indices, with the hypothesis that the six newly examined indices (DASH, AMED, HEI-2015, AHEI-2010, NRF9.3 and DQSJ) show inverse associations with mortality at least comparable to those of JDI12 and JFGST.

## Methods

### Study design and participants

We analysed data from the Takayama study. This prospective study was designed to investigate associations of diet and other lifestyle factors with the incidence of cancer, other chronic diseases and death. Details of the study design and the baseline characteristics of participants are described elsewhere^(32–34)^. The target population was all residents of Takayama City (Gifu Prefecture, Japan) aged 35 years or more, who numbered 37 287 as of 1 July 1992. A self-administered questionnaire was distributed to 36 990 residents in September 1992 after exclusion of those in hospital, away from the city, deceased or for other miscellaneous reasons. Of the 34 018 who returned the questionnaire, after excluding those who left many blanks or provided highly implausible responses^(33,34)^, the fixed cohort consisted of 31 552 individuals (participation rate, 85 %). We then excluded 2473 participants who reported a previous diagnosis of chronic heart disease, stroke or cancer at baseline, leaving 29 079 participants (13 355 men and 15 724 women), accounting for 78 % of all residents aged 35 years or more at the time (online Supplementary Figure 1). The Takayama study was approved by the ethical committee of Gifu University Graduate School of Medicine.

### Dietary assessment and calculation of diet quality indices

Dietary intake during the past year was assessed using a semi-quantitative FFQ at baseline. The FFQ asked about the intake frequency of foods, beverages and dishes (169 items) and usual portion size. Nutrient intake was estimated using the fifth revised and enlarged edition of the Japanese Standard Tables of Food Composition^(35)^. Intakes of *trans*-fat and added sugars were estimated using composition databases^(30,36)^. Dietary supplements were excluded from the calculation of nutrient intake. Details and validity of the FFQ were previously described^(37)^.

We calculated the eight diet quality indices, namely, the DASH^(38)^, AMED^(39)^, HEI-2015^(3)^, AHEI-2010^(4)^, NRF9.3^(29,30)^, DQSJ^(27)^, JFGST^(40)^ and JDI12^(23)^. The components and scoring criteria are shown in Table 1. For all indices, a higher score indicated a better-quality diet. The indices were calculated as in previous research^(23,27,28,40,41)^ (for details, see online Supplementary Methods section and Supplementary Table 1). To examine the validity of diet quality indices from the FFQ, we calculated correlations between seven indices from the FFQ and 12-d dietary records in thirty-seven adults; the JFGST was not calculated because the dietary records retained only food items, not dish names. The details of participants and dietary assessment are described elsewhere^(37)^. Spearman correlation coefficients between indices from the FFQ and 12-d dietary records in thirty-seven adults were 0·86 for the DQSJ, 0·79 for DASH, 0·74 for AHEI-2010, 0·72 for JDI12, 0·68 for NRF9.3, 0·59 for HEI-2015 and 0·52 for AMED. The validation sample showed higher scores and a larger sd of diet quality indices than the whole sample (online Supplementary Table 2).

**Table 1.** Components and scoring criteria of the eight diet quality indices used in this study Table 1 long description.

	Scoring criteria^*^	Favourable components^†^	Unfavourable components^‡^	Moderate components^§^
DASH	Group quintile	Fruit and fruit juices, vegetables, whole grains, low-fat dairy products, nuts and legumes	Red and processed meat, sugar-sweetened beverages, Na	–
AMED	Group median	Fruits, vegetables, whole grains, nuts, legumes, fish and ratio of MUFA to SFA	Red and processed meat	Alcohol
HEI-2015	Normative	Total fruits, whole fruits, total vegetables, greens and beans, whole grain, dairy products, total protein, seafood and plant protein foods, ratio of PUFA and MUFA to SFA	Refined grain, Na, SFA, added sugars	–
AHEI-2010	Normative^||^	Whole fruits, vegetables, nuts and legumes, PUFA, *n*-3 fatty acids, whole grain	Red and processed meat, sugar-sweetened beverages, Na, *trans*-fat	Alcohol
NRF9.3	Normative	Protein, dietary fibre, vitamins A, C, and D, Ca, Fe, potassium, Mg	Added sugars, saturated fats, Na	–
DQSJ	Group quartile	Fruits, vegetables, whole grains, dairy products, nuts, legumes, fish	Red and processed meat, sugar-sweetened beverages, Na	–
JFGST	Normative	–	Snack and alcoholic beverages	Grain dish, vegetable dish, fish and meat dish, fruits, milk
JDI12	Group median	Rice, miso, fish and shellfish, green and yellow vegetables, seaweed, pickles, green tea, soybeans and soybean-derived foods, fruits, mushrooms	Beef and pork, coffee	–

DASH, Dietary Approaches to Stop Hypertension; AMED, alternate Mediterranean diet; HEI-2015, Healthy Eating Index-2015; AHEI-2010, Alternate Healthy Eating Index-2010; NRF9.3, Nutrient Rich Food Index 9.3; DQSJ, Diet Quality Score for Japanese; JFGST, Japanese Food Guide Spinning Top; JDI12, twelve-item Japanese Diet Index.

* Criteria (cut-off values) of each component: normative (predefined servings or grams) or group intake.

† Higher intake receives a higher component score.

‡ Lower intake receives a higher component score.

§ Intake within the predefined range receives a higher component score.

|| Except for Na (scored using group decile).

### Follow-up and endpoints

The Takayama study collected information about death or moving away from Takayama City using residential registers or family registers. These data were collected from baseline (1 September 1992) to 1 October 2008. Cause of death was identified from death certificates provided by the Legal Affairs Bureau and classified according to the International Classification of Diseases, 10th Revision (ICD-10). The endpoints were mortality from all causes, all types of cancers (ICD-10 codes: C00–D48), CVD (I00–I99) and all other causes. We also analysed site-specific cancer mortality for major sites with at least 100 deaths in this cohort, including trachea, bronchus and lung cancer (C33–C34), stomach cancer (C16), colorectal cancer (C18–C21), liver cancer (C22) and pancreatic cancer (C25). Injury-related deaths (S00–T88) were analysed separately, with the hypothesis of no associations of diet quality indices with injury-related mortality. Within the study period, 1781 individuals (6·1 %) were censored on the day they left the city; if the leaving date was unknown (*n* 104), the date of last known residence was used. Mean (sd) of DQSJ for these 1781 individuals was 13·4 (3·7), similar to that of all other participants.

### Covariate measurement

The self-administered questionnaire at baseline included questions on sociodemographic factors, medical and reproductive history and lifestyle factors such as smoking, physical activity and sleep duration. Unmarked medical history was treated as ‘no’. Physical activity score (metabolic equivalent hours per week) was calculated by multiplying the time spent per week in various kinds of activities during the past year by their corresponding energy expenditure requirement, expressed as a metabolic equivalent, and summing these values. Missing responses for the time spent on physical activity were assigned with median values: 0 h/week for vigorous activity/physical labour and 0·5 h/week for moderate activity. Details and validity of this method have been reported elsewhere^(42)^. BMI (kg/m^2^) was calculated using self-reported height and weight.

### Statistical analysis

Means, sd and frequency of participant characteristics were described as quartiles of diet quality indices. The distributions of the eight indices were not markedly skewed (online Supplementary Figure 2). Therefore, we calculated Pearson correlation coefficients among diet quality indices. Additionally, Spearman correlation coefficients were calculated to examine associations of each diet quality index with intakes of selected foods and nutrients.

Cox proportional hazard models were used to calculate hazard ratios (HR) and 95% CI for mortality from all causes, CVD, total cancer, major cancer and other causes per 1 sd difference in each diet quality index^(43)^, under the hypothesis that each 1 sd increment in diet quality indices has beneficial effects. The proportional hazard assumption was confirmed by visual inspection of Schoenfeld residuals. The basic model was adjusted for sex and age at baseline. The adjusted model further included energy intake (continuous), a physical activity score (continuous), smoking (pack-year, continuous), BMI (continuous), hours of sleep per night (continuous: < 5 assigned 5, 6, 7, 8, 9, 10 < assigned 10), marital status (married or not), years of education (categories: less than 12, 12–14, 15 or more), history of hypertension (yes or no) and diabetes (yes or no), multivitamin use (yes or no) and menopause status (postmenopausal or premenopausal, women only, using sex and menopause status as an interaction term). BMI and sleep hours were included as quadratic terms in the model, considering their potential *U* or *J*-shaped associations with mortality^(44–46)^. Similar results were obtained when they were included as linear terms. To maximise data availability, we conducted ten rounds of multiple imputations for missing covariates (1·3% for marital status, 1·4 % for duration of education, 2·4% for menopause status, 4·1% for sleep duration, 5·6% for BMI, 6·1% for multivitamin use and 9·2% for pack-years of smoking) and then combined the estimates using the SAS PROC MI and MIANALYZE procedure.

We conducted several sensitivity analyses for all-cause, CVD and cancer mortality. Considering the possibility that associations of diet quality indices with mortality were not linear, we also examined HR across the quartiles of diet quality indices with tests of linear trend across increasing quartiles of indices by assigning the mean value to each category and treating them as continuous. Additionally, the HR for all-cause mortality per interquartile range were presented. To confirm the robustness of the associations, we also conducted the following sensitivity analyses for associations of diet quality indices with mortality: (1) exclusion of deaths in the first three years from baseline, to reduce the possibility of reverse causation; (2) analysis limited to participants without multivitamin use; (3) addition of alcohol intake (continuous, log-translation) as a covariate; (4) maximum follow-up period of 8 years (until 1 October 2000, approximately halfway through the total follow-up period) in consideration of a change in dietary intake after baseline; and (5) analysis limited to participants who reported all covariates (complete case analysis).

Stratified analysis was conducted to investigate whether HR were similar across various types of basic characteristics. Effect modification was tested using an interaction term of diet quality indices and the selected characteristics (multiplicative scale). The selected characteristics were sex, age (categorised as < 60 or ≥ 60 years), smoking status (never smoker or ever smoker) and BMI (underweight < 18·5 kg/m^2^, normal weight 18·5–24·9 kg/m^2^ or overweight ≥ 25 kg/m^2^). To examine whether observed associations were explainable by a single component in the indices, we examined associations of scores that removed each component from the diet quality indices with all-cause mortality, with adjustment for the removed component score.

Two-sided *P* values < 0·05 were regarded as statistically significant. We did not adjust for multiple comparisons because the primary aim was to present the HR for mortality across indices using the same analytical method and population, rather than to identify at least one statistically significant association among the eight indices. We used the SAS software, version 9.4. (SAS Institute) for data analysis.

## Results

### Study population and characteristics

This study included 13 355 men and 15 724 women at baseline. Table 2 shows the basic characteristics of the study population according to quartiles of the diet quality indices. For each of the eight diet quality indices, participants with higher scores were more likely to be older and use multivitamins and less likely to be current smokers. Higher scores were also associated with having a history of hypertension (except for JFGST) and a history of diabetes (except for HEI-2015). Associations with other characteristics varied among diet quality indices.

**Table 2.** Basic characteristics across the quartile of the 8 diet quality indices among Japanese adults (*n* 29 079)* Table 2 long description.

Score (mean, range)			DASH (21·8, 9**–**37)				AMED (3·8, 0**–**9)			
	All^†^		Q1		Q4		Q1		Q4	
Mean, range			16·9, 9–19		26·9, 25–37		1·6, 0–2		5·6, 5–9	
	*n*	%	*n*	%	*n*	%	*n*	%	*n*	%
Sex										
Men	13 355	45·9	3045	43·3	2887	42·2	2990	47·6	4464	45·6
Women	15 724	54·1	3987	56·7	3951	57·8	3296	52·4	5328	54·4
Marital status										
Married	23 724	81·6	5767	82·0	5508	80·5	4897	77·9	8223	84·0
Not married	4971	17·1	1197	17·0	1239	18·1	1308	20·8	1448	14·8
Duration of education										
≤ 11 years	17 778	61·1	3979	56·6	4315	63·1	3815	60·7	5981	61·1
12–14 years	8605	29·6	2373	33·7	1917	28·0	1896	30·2	2893	29·5
≥ 15 years	2288	7·9	593	8·4	519	7·6	483	7·7	795	8·1
Weight status										
Underweight	3895	13·4	808	11·5	943	13·8	915	14·6	1232	12·6
Normal weight	21 101	72·6	5134	73·0	4990	73·0	4553	72·4	7153	73·0
Overweight or obese	4083	14·0	1090	15·5	905	13·2	818	13·0	1407	14·4
Cigarette smoking										
Never smoker	13 802	47·5	3160	44·9	3727	54·5	2668	42·4	4974	50·8
Current smoker	8984	30·9	2752	39·1	1425	20·8	2340	37·2	2575	26·3
Former smoker	4273	14·7	729	10·4	1169	17·1	841	13·4	1576	16·1
History of hypertension (yes)	5254	18·1	1074	15·3	1398	20·4	1084	17·2	1820	18·6
History of diabetes (yes)	1217	4·2	175	2·5	434	6·3	243	3·9	455	4·6
Multivitamin use (yes)	8578	29·5	1892	26·9	2312	33·8	1647	26·2	3118	31·8
	Mean	sd	Mean	sd	Mean	sd	Mean	sd	Mean	sd
Age (years)	54·6	12·6	50·4	11·8	57·8	12·4	53·1	13·0	55·7	12·2
BMI (kg/m^2^)	22·2	2·9	22·4	2·9	22·1	2·8	22·1	2·9	22·3	2·9
Physical activity (MET-h/week)	22·6	35·8	23·1	36·3	21·2	33·7	20·8	34·5	23·7	35·8
Energy intake (kcal)	2357	863	2385	828	2330	897	2211	779	2499	923
Sleep duration (h/d)	7·2	1·0	7·1	1·0	7·2	1·1	7·2	1·1	7·2	1·0
	Median	25, 75 % tile	Median	25, 75 % tile	Median	25, 75 % tile	Median	25, 75 % tile	Median	25, 75 % tile
Alcohol intake (g/d), median	6·3	0·6, 35	9·3	1, 43	3·1	0, 25	3·9	0, 49	7·4	1·1, 25

Score, (mean, range)	HEI-2015 (55, 31**–**76)				AHEI-2010 (62, 31**–**96)				NRF9.3 (724, 163**–**878)			
	Q1		Q4		Q1		Q4		Q1		Q4	
Mean, range	49, 31–52		60, 57–76		53, 31–59		72, 66–96		648, 163–701		783, 762–878	
	*n*	%	*n*	%	*n*	%	*n*	%	*n*	%	*n*	%
Sex												
Men	3338	45·9	3339	45·9	3338	45·9	3339	45·9	3338	45·9	3339	45·9
Women	3931	54·1	3931	54·1	3931	54·1	3931	54·1	3931	54·1	3931	54·1
Marital status												
Married	5680	78·1	6047	83·2	5819	80·1	5989	82·4	5671	78·0	6008	82·6
Not married	1490	20·5	1134	15·6	1362	18·7	1171	16·1	1497	20·6	1166	16·0
Duration of education												
≤ 11 years	4584	63·1	4332	59·6	4217	58·0	4608	63·4	4691	64·5	4428	60·9
12–14 years	2032	28·0	2208	30·4	2372	32·6	1966	27·0	2001	27·5	2122	29·2
≥ 15 years	529	7·3	652	9·0	589	8·1	603	8·3	458	6·3	629	8·7
Weight status												
Underweight	1143	15·7	876	12·0	851	11·7	1040	14·3	1086	14·9	913	12·6
Normal weight	5097	70·1	5375	73·9	5265	72·4	5295	72·8	5180	71·3	5361	73·7
Overweight or obese	1029	14·2	1019	14·0	1153	15·9	935	12·9	1003	13·8	996	13·7
Cigarette smoking												
Never smoker	3101	42·7	3764	51·8	3095	42·6	3777	52·0	3117	42·9	3735	51·4
Former smoker	2626	36·1	1849	25·4	2906	40·0	1653	22·7	2693	37·0	1814	25·0
Current smoker	957	13·2	1177	16·2	854	11·7	1294	17·8	937	12·9	1217	16·7
History of hypertension (yes)	1232	16·9	1399	19·2	1195	16·4	1410	19·4	1239	17·0	1453	20·0
History of diabetes (yes)	302	4·2	354	4·9	209	2·9	418	5·7	257	3·5	350	4·8
Multivitamin use (yes)	1929	26·5	2478	34·1	2142	29·5	2181	30·0	1908	26·2	2405	33·1
	Mean	sd	Mean	sd	Mean	sd	Mean	sd	Mean	sd	Mean	sd
Age (years)	54·1	13·3	55·7	12·0	50·8	11·9	57·5	12·5	53·9	13·2	56·7	12·4
BMI (kg/m^2^)	22·1	3·0	22·3	2·8	22·4	3·0	22·1	2·8	22·1	2·9	22·3	2·8
Physical activity (MET-h/week)	20·5	34·9	23·3	35·5	24·5	38·0	21·6	34·6	21·0	35·5	23·1	36·1
Energy intake (kcal)	2228	798	2585	967	2634	956	2185	693	2281	852	2365	846
Sleep duration (h/d)	7·2	1·1	7·2	1·0	7·2	1·0	7·2	1·0	7·2	1·1	7·3	1·0
	Median	25, 75 % tile	Median	25, 75 % tile	Median	25, 75 % tile	Median	25, 75 % tile	Median	25, 75 % tile	Median	25, 75 % tile
Alcohol intake (g/d), median	6·2	0·5, 35	6·8	0·6, 39	8·2	0·6, 64	8·9	1·5, 22	7·4	0·6, 46	4·8	0·6, 28

Score (mean, range)	DQSJ (13·2, 0**–**26)				JFGST (34, 0**–**58)				JDI12 (6·0, 0**–**12)			
	Q1		Q4		Q1		Q4		Q1		Q4	
Mean, range	8·6, 0–10		17·6, 16–26		23, 0–30		44, 37–58		3·1, 0–4		9·2, 8–12	
	*n*	%	*n*	%	*n*	%	*n*	%	*n*	%	*n*	%
Sex												
Men	2984	47·1	3438	45·6	3338	45·9	3339	45·9	4006	45·6	2514	34·3
Women	3350	52·9	4106	54·4	3931	54·1	3931	54·1	4776	54·4	4824	65·7
Marital status												
Married	5135	81·1	6164	81·7	5924	81·5	5962	82·0	6956	79·2	5999	81·8
Not married	1136	17·9	1287	17·1	1256	17·3	1209	16·6	1724	19·6	1239	16·9
Duration of education												
≤ 11 years	3706	58·5	4759	63·1	4582	63	4357	59·9	4958	56·5	4906	66·9
12–14 years	2054	32·4	2084	27·6	2105	29	2167	29·8	2945	33·5	1908	26·0
≥ 15 years	503	7·9	606	8·0	481	6·6	648	8·9	765	8·7	416	5·7
Weight status												
Underweight	747	11·8	1009	13·4	902	12·4	973	13·4	746	8·5	545	7·4
Normal weight	4632	73·1	5545	73·5	5237	72·0	5339	73·4	6451	73·5	5319	72·5
Overweight or obese	955	15·1	990	13·1	1130	15·5	958	13·2	1145	13·0	1060	14·4
Cigarette smoking												
Never smoker	2679	42·3	3919	51·9	3188	43·9	3744	51·5	3904	44·5	4170	56·8
Former smoker	2610	41·2	1714	22·7	2684	36·9	1780	24·5	3226	36·7	1554	21·2
Current smoker	723	11·4	1358	18·0	939	12·9	1208	16·6	1157	13·2	948	12·9
History of hypertension (yes)	955	15·1	1511	20·0	1325	18·2	1321	18·2	1419	16·2	1438	19·6
History of diabetes (yes)	148	2·3	486	6·4	237	3·3	417	5·7	317	3·6	308	4·2
Multivitamin use (yes)	1624	25·6	2490	33·0	2169	29·8	2319	31·9	2314	26·3	2486	33·9
	Mean	sd	Mean	sd	Mean	sd	Mean	sd	Mean	sd	Mean	sd
Age (years)	50·5	12·1	57·8	12·2	53·7	12·6	56·0	12·4	52·3	12·8	57·0	12·0
BMI (kg/m^2^)	22·3	2·9	22·2	2·8	22·4	2·9	22·1	2·8	22·1	2·9	22·3	2·9
Physical activity (MET-h/week)	23·5	37·4	22·0	34·6	23·5	38·0	21·8	33·9	19·7	33·0	25·3	38·4
Energy intake (kcal)	2342	834	2371	875	2897	1036	2023	551	1906	624	2848	953
Sleep duration (h/d)	7·2	1·0	7·2	1·1	7·2	1·1	7·2	1·0	7·2	1·0	7·2	1·1
	Median	25, 75 % tile	Median	25, 75 % tile	Median	25, 75 % tile	Median	25, 75 % tile	Median	25, 75 % tile	Median	25, 75 % tile
Alcohol intake (g/d), median	10·6	0·9, 46	4·2	0·5, 28	25·5	1·5, 64	2·4	0, 13	5·3	0·6, 35	3·8	0·6, 27

DASH, Dietary Approaches to Stop Hypertension; AMED, alternate Mediterranean diet; HEI-2015, Healthy Eating Index-2015; AHEI-2010, Alternate Healthy Eating Index-2010; NRF9.3, Nutrient Rich Food Index 9.3; DQSJ, Diet Quality Score for Japanese; JFGST, Japanese Food Guide Spinning Top; JDI12, twelve-item Japanese Diet Index; MET, metabolic equivalent; Med, median; Q1, Quartile 1 (25 % tile); Q3, Quartile 3 (75 %tile).

* The participants were first categorised into four groups based on sex-specific quartiles of the indices and then analysed together.

† 28 695 for marital status; 28 671 for duration of education; 27 309 for multivitamin use; 27 896 for sleep duration; 27 438 for BMI.

As shown in Table 3, except for almost zero correlation between JFGST and JDI12, the eight diet quality indices showed correlations with each other that ranged from very low (Pearson’s correlation coefficient = 0·08 between AMED and JFGST) to strong (0·80 between the DASH and DQSJ). Correlations with intakes of nutrients and food groups were partially consistent across the diet quality indices, although some differences were observed (online Supplementary Figure 3). All eight indices were positively correlated with intakes of fibre (point estimate of Spearman correlation coefficients range: 0·34–0·53), Mg (0·26–0·55), Fe (0·26–0·55), fruits (0·20–0·65), Ca (0·24–0·51), vitamin C (0·23–0·54) and protein (0·15–0·43). Although Na was included as an unfavourable component of the DASH, HEI-2015, AHEI-2010, NRF9.3 and DQSJ, Na intake was positively correlated with these indices (0·09–0·35), as well as the other three (0·16 for JFGST, 0·46 for AMED and 0·47 for JDI12).

**Table 3.** Pearson correlation coefficients between diet quality indices in Japanese adults (*n* 29 079) Table 3 long description.

	DASH	AMED	HEI-2015	AHEI-2010	NRF9.3	DQSJ	JFGST	JDI12
DASH	–	–	–	–	–	–	–	–
AMED	0·47	–	–	–	–	–	–	–
HEI-2015	0·47	0·50	–	–	–	–	–	–
AHEI-2010	0·65	0·50	0·37	–	–	–	–	–
NRF9.3	0·39	0·37	0·41	0·34	–	–	–	–
DQSJ	0·80	0·58	0·45	0·62	0·53	–	–	–
JFGST	0·31	0·08	0·17	0·40	0·35	0·28	–	–
JDI12	0·38	0·50	0·38	0·31	0·35	0·42	–0·03	–

DASH, Dietary Approaches to Stop Hypertension; AMED, alternate Mediterranean diet; HEI-2015, Healthy Eating Index-2015; AHEI-2010, Alternate Healthy Eating Index-2010; NRF9.3, Nutrient Rich Food Index 9.3; DQSJ, Diet Quality Score for Japanese; JFGST, Japanese Food Guide Spinning Top; JDI12, twelve-item Japanese Diet Index.

### Diet quality indices and mortality

The mean duration of follow-up was 14·1 years. During 410 352 person-years of follow-up, 5339 deaths (including 1678 from CVD and 1620 from cancer) were recorded. For each 1-sd higher diet quality index, the HR (95 % CI) for all-cause mortality were 0·90 (0·87, 0·93) for AHEI-2010, 0·92 (0·89, 0·95) for DQSJ, 0·93 (0·91, 0·96) for NRF9.3, 0·94 (0·92, 0·97) for AMED and DASH, 0·94 (0·91, 0·97) for JFGST, 0·94 (0·91, 0·98) for JDI12 and 0·97 (0·94, 0·996) for HEI-2015 in the fully adjusted model (Table 4). Similar results were obtained for mortality from CVD and other causes with wider ranges of 95 % CI. In contrast, cancer mortality was associated with none of the indices except NRF9.3 (HR: 0·95, 95 % CI: 0·91, 0·999). Associations with site-specific cancer mortality were generally inconsistent across indices (online Supplementary Table 3). During follow-up, 370 deaths from injury were observed; none of the indices was associated with injury-related mortality, with HR (95 % CI) ranging from 0·95 (0·84, 1·07) for DQSJ to 1·00 (0·90, 1·12) for AHEI-2010.

**Table 4.** Hazard ratios of mortality of all causes, CVD, cancer and other causes for 1 sd increments in the eight diet quality indices among Japanese adults (*n* 29 079) Table 4 long description.

	Model 1^*^		Model 2^†^	
	HR	95 % CI	HR	95 % CI
All causes	*n* of deaths = 5339			
DASH	0·93	0·91, 0·96	0·94	0·92, 0·97
AMED	0·91	0·89, 0·94	0·94	0·92, 0·97
HEI-2015	0·94	0·92, 0·97	0·97	0·94, 0·996
AHEI-2010	0·89	0·87, 0·92	0·90	0·87, 0·93
NRF9.3	0·91	0·89, 0·93	0·93	0·91, 0·96
DQSJ	0·91	0·88, 0·93	0·92	0·89, 0·95
JFGST	0·97	0·94, 0·996	0·94	0·91, 0·97
JDI12	0·91	0·88, 0·93	0·94	0·91, 0·98
CVD	*n* of deaths = 1678			
DASH	0·90	0·86, 0·95	0·91	0·86, 0·95
AMED	0·91	0·86, 0·95	0·95	0·90, 0·998
HEI-2015	0·95	0·90, 0·99	0·98	0·93, 1·03
AHEI-2010	0·88	0·84, 0·93	0·88	0·84, 0·93
NRF9.3	0·91	0·87, 0·95	0·93	0·89, 0·97
DQSJ	0·88	0·84, 0·93	0·89	0·85, 0·94
JFGST	0·98	0·94, 1·03	0·93	0·88, 0·98
JDI12	0·87	0·83, 0·92	0·92	0·87, 0·98
Cancer	*n* of deaths = 1620			
DASH	1·02	0·97, 1·07	1·03	0·98, 1·09
AMED	0·97	0·93, 1·02	0·99	0·94, 1·04
HEI-2015	0·97	0·92, 1·01	0·98	0·93, 1·03
AHEI-2010	0·98	0·93, 1·03	0·99	0·94, 1·05
NRF9.3	0·94	0·90, 0·98	0·95	0·91, 0·999
DQSJ	1·00	0·95, 1·05	1·01	0·96, 1·07
JFGST	0·98	0·93, 1·03	0·99	0·93, 1·04
JDI12	0·99	0·95, 1·04	1·02	0·96, 1·08
Other causes	*n* of deaths = 2041			
DASH	0·91	0·86, 0·95	0·92	0·87, 0·96
AMED	0·88	0·84, 0·92	0·91	0·87, 0·95
HEI-2015	0·92	0·89, 0·97	0·96	0·91, 1·000
AHEI-2010	0·85	0·81, 0·89	0·85	0·81, 0·90
NRF9.3	0·90	0·86, 0·93	0·92	0·89, 0·96
DQSJ	0·87	0·83, 0·91	0·88	0·84, 0·92
JFGST	0·96	0·92, 1·002	0·92	0·88, 0·97
JDI12	0·88	0·85, 0·93	0·92	0·87, 0·97

DASH, Dietary Approaches to Stop Hypertension; AMED, alternate Mediterranean diet; HEI-2015, Healthy Eating Index-2015; AHEI-2010, Alternate Healthy Eating Index-2010; NRF9.3, Nutrient Rich Food Index 9.3; DQSJ, Diet Quality Score for Japanese; JFGST, Japanese Food Guide Spinning Top; JDI12, twelve-item Japanese Diet Index.

The values of sd for each score are presented in online Supplementary Table 2.

* Adjusted for age and sex.

† Adjusted for age, sex, total energy intake (continuous), BMI (continuous, as quadratic term), physical activity (continuous), smoking (packs per year, continuous), education (less than 12 years, 12–14 years, 15 years or more), marital status, sleep duration (continuous, as quadratic term), history of hypertension (yes or no), history of diabetes (yes or no), multivitamin use (yes or no) and menopause status (yes or no, only for women).

### Sensitivity analysis

Similar associations were obtained when analysing using the interquartile range or the quartiles of diet quality indices instead of 1 sd of diet quality indices. The HR for all-cause mortality per interquartile range were as follows: 0·87 (0·84, 0·90) for AHEI-2010, 0·89 (0·85, 0·93) for DQSJ, 0·91 (0·86, 0·96) for JDI12, 0·92 (0·90, 0·95) for NRF9.3, 0·92 (0·88, 0·96) for JFGST, 0·93 (0·89, 0·96) for DASH, 0·93 (0·90, 0·96) for AMED and 0·96 (0·92, 0·99) for HEI-2015. The HR (95 % CI) for all-cause mortality in the highest quartiles of diet quality indices compared with the lowest quartiles were 0·78 (0·82, 0·85) for AHEI-2010, 0·82 (0·75, 0·89) for DQSJ, 0·84 (0·78, 0·91) for NRF9.3, 0·87 (0·80, 0·94) for JFGST, 0·87 (0·80, 0·95) for DASH and JDI12, 0·88 (0·81, 0·95) for AMED and 0·93 (0·86, 1·004) for HEI-2015 (online Supplementary Table 4). For AHEI-2010 and DQSJ, higher quartile appeared to be gradually associated with lower HR: 0·93 (0·85, 1·01) for Q2, 0·88 (0·81, 0·95) for Q3, 0·78 (0·72, 0·85) for Q4 in AHEI-2010 and 0·89 (0·82, 0·96) for Q2, 0·86 (0·79, 0·94) for Q3 and 0·82 (0·75, 0·89) for Q4 in DQSJ. The HR of NRF9.3 for cancer mortality were 0·79 (0·68, 0·91) in Q2, 0·85 (0·74, 0·97) in Q3 and 0·89 (0·78, 1·01) in Q4 (online Supplementary Table 5). Sensitivity analysis under various assumptions showed that HR for all causes, CVD and cancer per 1 sd difference of each diet quality index were similar to those in the main analysis (online Supplementary Table 6).

**Figure 1.**
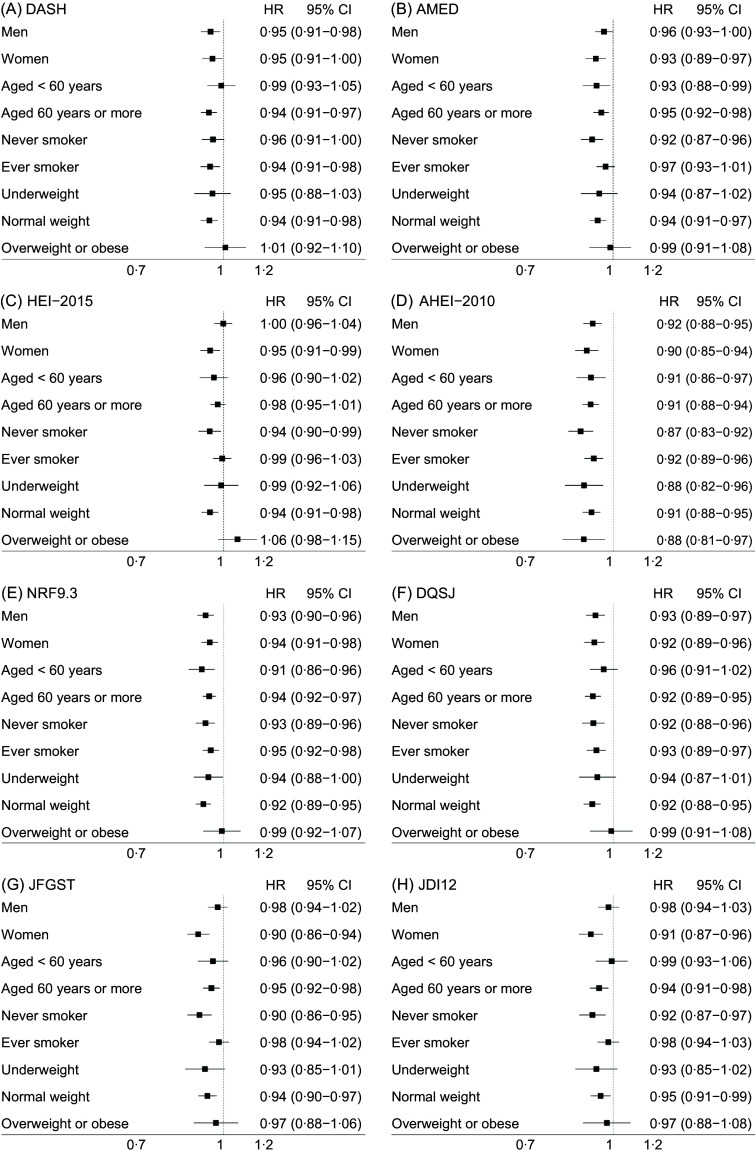
Figure 1 long description. Hazard ratios (HR) on all-cause mortality per 1 sd higher score of each diet quality index among Japanese adults (*n* 29 079), stratified by basic characteristics (sex, age, smoking status and weight status)^a^. DASH, Dietary Approaches to Stop Hypertension; AMED, alternate Mediterranean diet; HEI-2015, Healthy Eating Index-2015; AHEI-2010, Alternate Healthy Eating Index-2010; NRF9.3, Nutrient Rich Food Index 9.3; DQSJ, Diet Quality Score for Japanese; JFGST, Japanese Food Guide Spinning Top; JDI12, twelve-item Japanese Diet Index. ^a^The horizontal axis for HR is scaled logarithmically. Cox proportional hazards models to estimate HR and 95 % CI were adjusted for age, sex, total energy intake (continuous), BMI (continuous, as quadratic term), physical activity (continuous), smoking (packs per year, continuous), education (less than 12 years, 12**–**14 years, 15 years or more), marital status, sleep duration (continuous, as quadratic term), history of hypertension (yes or no), history of diabetes (yes or no), multivitamin use (yes or no), menopause status (yes or no, only for women). Participant number and cases were 13 355 and 2901 for men, 15 724 and 2438 for women, 19 128 and 1212 for participants younger than 60 years, 9951 and 4127 for participants 60 years old or more, 13 802 and 1976 for never smokers, 13 257 and 2785 for ever smokers, 3895 and 1416 for underweight, 21 101 and 3343 for normal weight and 4083 and 580 for overweight. Interactions were observed in HEI-2015 with sex (*P* = 0·02) and overweight or obesity status (*v*. normal and underweight, *P* = 0·01), JFGST with smoking status (*P* = 0·053) but not with sex (*P* = 0·20) and JDI12 with sex (*P* = 0·01) and smoking status (*P* = 0·048).

When stratified by basic characteristics, the AHEI-2010 showed significant associations with all-cause mortality across all strata (Figure 1). Except for AHEI-2010, no indices were associated with all-cause mortality among individuals with overweight or obesity, but interactions were significant only for HEI-2015. HEI-2015 and JDI12 showed interactions with several characteristics, including sex (weaker associations in men than in women). Similar associations were obtained for HR for CVD (online Supplementary Figure 4). Only NRF9.3 was associated with cancer mortality in some strata (men, ever smokers and age below 60 years) (online Supplementary Figure 5).

When one component was omitted from the diet quality indices, the point estimates of HR for all-cause mortality per 1 sd difference did not change markedly, ranging from 0·89 to 0·94 for AHEI-2010, 0·90 to 0·94 for NRF9.3, 0·92 to 0·95 for DQSJ, 0·93 to 0·96 for AMED, 0·94 to 0·96 for JFGST, 0·93 to 0·97 for JDI12, 0·94 to 0·97 for DASH and 0·95 to 0·98 for HEI-2015 (online Supplementary Figure 6).

## Discussion

In this study in Japan, all eight diet quality indices were associated with lower all-cause mortality. The HR (95 % CI) for all-cause mortality per 1 sd higher index ranged from 0·90 (0·87, 0·93) for AHEI-2010 to 0·97 (0·94, 0·996) for HEI-2015. These findings suggest that these eight indices are beneficial for longevity but that they differ in the strength of their associations.

Previous studies have examined the associations of the Japanese Diet Index and JFGST with all-cause mortality in Japan^(9,10,13)^. Point estimates of HR for the highest *v*. lowest quartiles were 0·91 for the JFGST^(13)^ and 0·86^(9)^ and 0·91^(10)^ for the Japanese Diet Index. These are similar to our study (0·87 for both indices), despite some differences in how scores were calculated. In contrast, another study did not observe an association of AHEI-2010 with CVD mortality in Japan^(47)^, which contradicts our results. This difference may be partly explained by differences in the FFQ used (40 items in the previous study^(47)^ *v*. 169 in the present study). In the present study, AHEI-2010 showed a high correlation between the FFQ and 12-d dietary records, whereas the validity of the FFQ for assessing AHEI-2010 was not examined in the previous study. The lack of research on associations of Western-origin indices with mortality in Japan might reflect challenges in assessing these indices using the FFQ employed in Japanese cohort studies. FFQ with a small number of food items^(10,11,47)^ may focus primarily on commonly consumed foods, thereby potentially omitting foods that are rarely consumed in Japan but included in Western-origin indices, such as whole grains and nuts.

The DASH, Mediterranean diet, Healthy Eating Index and Alternate Healthy Eating Index have been generally associated with lower all-cause mortality in not only Western but also non-Western countries, including China^(19)^, Iran^(17)^ and Chinese adults in Singapore^(48)^. The Western-origin indices include broader food groups and nutrients rather than specific food items, which may contribute to their generalizability to non-Western countries. However, given that meta-analyses have reported substantial heterogeneity in associations of these indices with all-cause mortality^(6–8)^, the effect sizes may vary by index and region. A meta-analysis suggested that the association of Mediterranean diet scores with all-cause mortality may be weaker in non-Mediterranean countries, and the HR of 0·92 per 2-point increments in non-Mediterranean countries was similar to our finding of an HR of 0·94 per 1 sd (1·6 points)^(6)^. Similarly, a meta-analysis showed that the Healthy Eating Index may have weaker associations with all-cause mortality in non-US countries, and HR were similar to those in our study and in non-US countries (0·93 for Q4 *v*. Q1 in the present study and 0·90 for the highest *v*. lowest categories in a meta-analysis)^(7)^. Although region-specific HR have not reported for the Alternate Healthy Eating Index or DASH, the HR in our study were similar to the estimates from the meta-analysis for the Alternate Healthy Eating Index but somewhat higher than those for DASH^(8)^. Regarding NRF9.3, similar associations to our present findings were observed in a study in the Netherlands^(15)^. Taken together, the most appropriate index may vary by region, warranting studies to identify optimal choices in each region or country.

When stratified by basic characteristics, AHEI-2010 showed the most consistent associations with lower all-cause mortality across all strata. Among individuals with overweight or obesity, except for AHEI-2010, all indices showed null associations with all-cause mortality. One possible explanation is that individuals with obesity may overestimate their diet quality, as they tend to underreport their intake of fatty or sugary foods^(49)^. In addition, other factors such as excessive energy intake, obesity itself and obesity-related diseases may play a more important role in all-cause mortality among individuals with obesity, although we adjusted for BMI, energy intake and history of self-reported diabetes and blood pressure. It is unclear why only AHEI-2010 was associated with all-cause mortality among individuals with overweight or obesity. However, compared with the other indices, the AHEI-2010 includes a larger proportion of unfavourable components (four unfavourable, one moderate and six favourable components; Table 1), which may partly explain the observed associations in this group. Further, several indices had weaker associations with all-cause mortality in men than in women. Further research is needed to explore optimal diet quality indices according to individual characteristics.

In our study, except for NRF9.3, none of the diet quality indices examined was associated with cancer mortality. These null associations may be at least partly due to heterogeneity in the association of dietary intake with cancer across sites and types of cancer. Previous studies also observed that, compared with mortality from all causes and CVD, cancer mortality was only weakly or not significantly associated with diet quality indices in Japan^(9,11–14,47,50)^ and other non-Western countries^(17,19,48)^. Regarding NRF9.3, we observed higher total cancer mortality in the lowest quartile. Additionally, our results suggest inverse associations with mortality from lung, colorectal and pancreatic cancer, although these associations were non-significant due to the small number of deaths. The NRF9.3 may capture suboptimal intakes of nutrients that may contribute to increasing mortality from or risks of lung, colorectal and pancreatic cancer, such as retinol, vitamin C, fibre and SFA^(51–53)^.

We found moderate to strong correlations between Western-origin indices and DQSJ (Spearman’s coefficients ranged from 0·3 to 0·8), whereas the JDI12 and JFGST showed none or weak to moderate associations with the other indices (–0·0 to 0·5). Correlations with intakes of food groups and nutrients also somewhat differed across indices. Nevertheless, all diet quality indices showed beneficial associations with all-cause mortality. Therefore, these indices may capture somewhat different aspects of diet that are beneficial for longevity in the Japanese population. Additionally, the results of omitting one component from the indices suggest that no single component is indispensable in assessing diet quality.

We examined eight diet quality indices using the same dataset and statistical methods; nevertheless, there are no established criteria determining the superiority of any particular index, and we did not statistically compare associations with mortality across indices as in previous studies^(4,17,19–21,43,48)^. Although it was difficult to determine a clear superiority of particular indices, associations tended to be strongest for the AHEI-2010, followed by the DQSJ, and weakest for the HEI-2015. The AHEI-2010 and DQSJ were developed relatively recently based on evidence of associations of diet with mortality and risks of chronic diseases, which may explain the relatively strong associations with mortality. Nevertheless, the HR were not markedly different between these indices and the existing Japanese indices (JFGST and JDI12). One possible explanation is that both the AHEI-2010 and DQSJ include components that were rarely consumed in this population (e.g., whole grains), which may have limited their ability to capture diet quality in this setting. On the other hand, the HEI-2015 is a measure of adherence to dietary guidelines for Americans that are based on food intake data from the US population^(54)^, which may lead to a lesser ability to assess healthy dietary patterns in Japan compared with the other indices. The comparable estimates for multiple indices in this study may be informative for future research and practical settings, such as interventions, dietary counselling and policy making. In such contexts, it may be appropriate to select indices that show stronger associations with health outcomes. Further research should still examine multiple diet quality indices simultaneously to identify optimal indices regarding health outcomes in Japan, as well as other countries^(16,43)^.

The main strengths of this study are the assessment of various types of diet quality indices using the same analysis methods and participation by a high proportion of the entire population of Takayama City, with a low rate of loss during follow-up. Several limitations should be mentioned. First, dietary intake and other covariates were assessed only at baseline. Although we confirmed that associations of diet quality indices with mortality did not substantially differ between the two follow-up periods (8 and 16 years), subsequent changes in dietary intake and other covariates during follow-up may have led to the under- or overestimation of associations. Second, we cannot rule out the possibility of residual or unadjusted confounders. For example, although we carefully adjusted for education level and lifestyle factors such as smoking, physical activity and sleep duration, we did not assess economic status. Third, we conducted multiple statistical analyses, and some significant associations may accordingly have arisen by chance. Also, our sample size might have been insufficient for detecting interactions of sociodemographic factors and diet quality indices on mortality. However, since our aim was not to detect these interactions but to examine associations of diet quality indices with mortality, we assessed the consistency of the observed associations across various characteristics and under different assumptions. Given these limitations and the restricted study area, further research is needed to confirm the observed associations in other Japanese populations.

### Conclusions

This study in a Japanese population showed that higher scores on eight diet quality indices (DASH, AMED, HEI-2015, AHEI-2010, NRF9.3, DQSJ, JFGST and JDI12) were associated with lower all-cause and CVD mortality but were not consistently associated with cancer mortality. Beneficial associations of the indices cannot be explained by a single food or nutrient component, suggesting the importance of assessing overall dietary patterns, rather than a single food or nutrient. Although it is difficult to determine a clear superiority of any particular index, the AHEI-2010 showed relatively strong and consistent associations, which were followed by the DQSJ, whereas the HEI-2015 appeared to have relatively weaker associations in this Japanese population. Our results may be informative for future research and practical settings, such as interventions, dietary counselling and policy making. Nevertheless, further research is needed to examine the reproducibility of these findings in other Japanese populations, as well as the associations of these indices with other health outcomes.

## Supporting information

10.1017/S0007114525106077.sm001 Oono et al. supplementary material Oono et al. supplementary material

## Supplementary material

For supplementary material/s referred to in this article, please visit https://doi.org/10.1017/S0007114525106077.

## Figure 1 Long description

The table presents hazard ratios (HR) and 95 percent confidence intervals (CI) on all-cause mortality per one standard deviation higher score of various diet quality indices among Japanese adults, stratified by sex, age, smoking status, and weight status. The indices include DASH, AMED, HEI-2015, AHEI-2010, NRF9.3, DQSJ, JFGST, and JDI12. The table has eight columns: one for the diet quality index names, one for hazard ratios, one for 95 percent confidence intervals, and five for different stratifications: men, women, aged less than 60 years, aged 60 years or more, never smoker, ever smoker, underweight, normal weight, and overweight or obese. Each row provides the HR and 95 percent CI for each stratification. Notable trends include generally lower hazard ratios for higher diet quality scores across most stratifications, indicating a potential inverse association between diet quality and mortality risk.

Navigate back to Figure 1.

## Table 1 Long description

The table presents a comparison of the components and scoring criteria for eight diet quality indices: DASH, AMED, HEI-2015, AHEI-2010, NRF9.3, DQSJ, JFGST, and JDI12. The table has eight rows and four columns. The columns are labeled 'Scoring criteria', 'Favourable components', 'Unfavourable components', and 'Moderate components'. Each row corresponds to one of the diet quality indices. For DASH, the scoring criteria is group quintile, with favourable components including fruit and fruit juices, vegetables, whole grains, low-fat dairy products, nuts, and legumes, and unfavourable components including red and processed meat, sugar-sweetened beverages, and sodium. AMED uses group median as the scoring criteria, with favourable components such as fruits, vegetables, whole grains, nuts, legumes, fish, and the ratio of monounsaturated fatty acids to saturated fatty acids, and unfavourable components including red and processed meat, with alcohol as a moderate component. HEI-2015 uses normative scoring criteria, with favourable components like total fruits, whole fruits, total vegetables, greens and beans, whole grains, dairy products, total protein, seafood and plant protein foods, and the ratio of polyunsaturated fatty acids and monounsaturated fatty acids to saturated fatty acids, and unfavourable components including refined grain, sodium, saturated fatty acids, and added sugars. AHEI-2010 also uses normative scoring criteria, with favourable components such as whole fruits, vegetables, nuts and legumes, polyunsaturated fatty acids, n-3 fatty acids, and whole grains, and unfavourable components including red and processed meat, sugar-sweetened beverages, sodium, and trans-fat, with alcohol as a moderate component. NRF9.3 uses normative scoring criteria, with favourable components like protein, dietary fibre, vitamins A, C, and D, calcium, iron, potassium, and magnesium, and unfavourable components including added sugars, saturated fats, and sodium. DQSJ uses group quartile as the scoring criteria, with favourable components such as fruits, vegetables, whole grains, dairy products, nuts, legumes, and fish, and unfavourable components including red and processed meat, sugar-sweetened beverages, and sodium. JFGST uses normative scoring criteria, with unfavourable components including snack and alcoholic beverages, and moderate components such as grain dish, vegetable dish, fish and meat dish, fruits, and milk. JDI12 uses group median as the scoring criteria, with favourable components including rice, miso, fish and shellfish, green and yellow vegetables, seaweed, pickles, green tea, soybeans and soybean-derived foods, fruits, and mushrooms, and unfavourable components including beef and pork, and coffee.

Navigate back to Table 1.

## Table 2 Long description

The table presents a comparison of basic characteristics across quartiles of eight diet quality indices among Japanese adults, with a sample size of 29,079. It includes data for 13,355 men and 15,724 women. The table is organized into columns for different diet quality indices and rows for various characteristics such as age, sex, marital status, education, occupation, smoking status, alcohol consumption, multivitamin use, history of hypertension, and history of diabetes. Notable trends include higher scores associated with older age, multivitamin use, and a history of hypertension and diabetes, except for specific indices. The table highlights variations in associations with other characteristics among the different diet quality indices.

Navigate back to Table 2.

## Table 3 Long description

A table with eight rows and eight columns displaying Pearson correlation coefficients between various diet quality indices in Japanese adults. The rows and columns are labeled with the names of the diet quality indices: DASH, AMED, HEI-2015, AHEI-2010, NRF9.3, DQSJ, JFGST, and JDI12. The table shows correlation values ranging from very low to strong, with notable values including 0.80 between DASH and DQSJ, and almost zero correlation between JFGST and JDI12. The table provides insights into the relationships between different diet quality indices.

Navigate back to Table 3.

## Table 4 Long description

The table presents hazard ratios (HR) and 95 percentage confidence intervals (CI) for mortality from all causes, cardiovascular disease (CVD), cancer, and other causes associated with one standard deviation increments in eight diet quality indices among Japanese adults. The table includes data for 29,079 participants with a mean follow-up duration of 14.1 years. It features two models with HR and CI values for each diet quality index. The indices include DASH, AMED, HEI-2015, AHEI-2010, NRF9.3, DQSJ, JFGST, and JDI12. The table is divided into four sections based on the cause of mortality: all causes, CVD, cancer, and other causes. Each section lists the HR and CI for each diet quality index. Notable trends include lower hazard ratios for all-cause mortality with higher diet quality scores, particularly for AHEI-2010 and DQSJ. Cancer mortality shows less consistent associations with the diet quality indices compared to other causes of mortality.

Navigate back to Table 4.
