# Supplementary material for: Associations of diet quality indices with all-cause and cause-specific mortality among Japanese adults in the Takayama study
Source: Br J Nutr. 2025 Dec 29;135(7):737–48. doi: 10.1017/S0007114525106077 (PMC13246593; doi:10.1017/S0007114525106077)
Supplement: Oono et al. supplementary material [file S0007114525106077sup001.pdf]

Supplemental Materials

Title: Associations of Diet Quality Indices with All-cause and Cause-specific Mortality Among Japanese Adults in the Takayama Study

Authors: Fumi Oono[1,2], Keiko Wada[1], Michiyo Yamakawa[1], Masaaki Sugino[1], Tomoka Mori[1], Shino Oba[3], Kentaro Murakami[4], Chisato Nagata[1]

Author affiliations:

[1] Department of Epidemiology and Preventive Medicine, Graduate School of Medicine, Gifu University, Gifu, Japan

[2] Department of Social and Preventive Epidemiology, Division of Health Sciences and Nursing, Graduate School of Medicine, The University of Tokyo, Tokyo, Japan

[3] Graduate School of Health Sciences, Gunma University, Maebashi, Gunma, Japan

[4] Department of Social and Preventive Epidemiology, School of Public Health, The University of Tokyo, Tokyo, Japan

Corresponding Author:

Fumi OONO, PhD

Department of Epidemiology and Preventive Medicine, Graduate School of Medicine, Gifu University, 1-1 Yanagido, Gifu 501-1194, Japan.

Tel: +81-58-230-6412

Email: fumioono@m.u-tokyo.ac.jp

Supplemental methods

Calculation of the 8 diet quality indices

For all indices, higher scores indicate a better diet quality. For the DASH, AMED, and DQSJ, whole grain intake was calculated as the dry-weight of whole grain, such as brown rice before cooking, or whole grain flour in bread. This estimation was derived using information on ingredients and weight change in cooking in Standard Tables of Food Composition in Japan [(1)].

1. Dietary Approaches to Stop Hypertension score (DASH)

   We calculated the Fung's DASH score [(2)] because of its wide use in previous studies.[(3; 4; 5)]. DASH scores range from 8 to 40. The DASH comprises eight components, each given 1–5 points based on the quintile of intake of each component in the study. The highest quintile is assigned 5 points for five components (fruits and fruit juice, vegetables, whole grains, nuts and legumes, and reduced-fat dairy products), while the lowest quintile is assigned 5 points for three components (red and processed meat, sugar-sweetened beverages, and sodium). Scores were calculated using sex-specific quintile intakes of components (g/1,000 kcal) in this population. A large proportion of participants reported that they did not consume whole grain (95.1%) and reduced-fat dairy products (41.9%). Therefore, 1 point was assigned to non-consumers, and 2–5 points were assigned to consumers by quartile of intake among consumers. Fruits included all fruits and fruit juice; vegetables excluded potatoes and legumes; and low-fat dairy included all reduced-fat dairy products such as milk and yogurt. Other definitions were the same as for the DQSJ.

2. Alternate Mediterranean diet score (AMED)

   Among the various type of the Mediterranean diet, the alternate Mediterranean diet score (AMED) [(6)] was chosen due to its wide use in studies.[(3; 4; 5)] The AMED consists of nine components, each given 0 or 1 point based on median intake of each component (g/1,000 kcal) except for alcohol. Participants gained 1 point for intakes of seven components (fruits and fruit juice, vegetables except for potatoes, whole grains, nuts, legumes, fish, and the ratio of monosaturated fatty acids to saturated fatty acids) above median. Participants gained 1 point for intakes of red and processed meat below the median. Participants gained 1 point if their alcohol intake was 5–20 g ethanol/day. Because more than half of the participants did not consume whole grain during the study, non-consumers received 0 points, and consumers received 1 point.

3. Healthy Eating Index (HEI)-2015

HEI-2015 assesses adherence to the 2015–2020 Dietary Guidelines for Americans [7]. Scores range from 0 to 100. The HEI-2015 was calculated based on a previous study using a diet history questionnaire in Japan [8] and the 2011–2012 Food Patterns Equivalents Database[9]. The maximum points and the refence intakes of each component were as follows: total fruits, 5 points for ≥0.8 cup eq/1,000 kcal; whole fruits, 5 points for ≥0.4 cup eq/1,000 kcal; total vegetables, 5 points for ≥1.1 cup eq/ 1,000 kcal; greens and beans, 5 points for ≥0.2 cup eq/1,000 kcal; whole grain, 10 points for ≥1.3 cup eq/1,000 kcal; dairy, 10 points for ≥1.3 cup eq/1,000 kcal; total protein foods, 5 points for ≥2.5 oz eq/1,000 kcal; seafood and plant protein, 5 points for ≥0.8 cup eq/1,000 kcal; fatty acids (ratio of the sum of polyunsaturated and monounsaturated fatty acids to saturated fatty acids), 10 points for ≥2.5; refined grain, 10 points for ≤1.8 oz eq/1,000 kcal; sodium, 10 points for ≤1.1 g/1,000 kcal; added sugars, 10 points for ≤6.5% of total energy; and saturated fats, 10 points for ≤8% of total energy. For nine favorable components, the standards for minimum score (0 points) were 0-unit eq (non-consumers). For four unfavorable components, standards for minimum score (0 points) were ≥4.3 oz eq/1,000 kcal for refined grain, ≥2.0 g/1,000 kcal for sodium, ≥26% of energy for added sugar, and ≥16% of energy for SFA.

4. Alternate Healthy Eating Index (AHEI)-2010

The AHEI–2010 scores range from 0 to 110 [10]. The standards for the minimum (0 points) and maximum scores (10 points) per day were 0 and ≥5 sv for vegetables, 0 and ≥4 sv for fruits, 0 and ≥75 g for whole grains, 0 and ≥1 sv for nuts and legumes, 0 and 250 mg for long-chain (n-3) fats, ≤2 and ≥10% of energy for PUFA, ≥1 and 0 sv for SSBs and fruit juice, ≥1.5 and 0 sv for red and processed meat, ≥4 and 0% of energy for trans-fat, ≥highest decile and lowest decile for sodium. For alcohol, the minimum scores were assigned for ≥2.5 drinks for women and ≥3.5 drinks for men, and the maximum scores were assigned for 0.5–1.5 drinks. Nondrinkers received a score of 2.5. In this study, fruit did not include 100% fruit juice, and vegetables included dark green vegetables, tomatoes, orange vegetables, other vegetables, fresh green beans, and pickled vegetables. One serving of fruits was assumed to be 80 g of fruits and 1 serving of vegetables was assumed to be 80 g of vegetables, based on a previous study[11]. For red and processed meat, one serving was 113 g of unprocessed red meat and 42.5g of processed meat. One alcoholic drink of alcohol was assumed to be 14 g of ethanol using the Dietary Guidelines for Americans 2015–2020 [12]. No energy adjustment was performed for component intake (except for PUFA and trans-fat) following the original paper [10].

5. Nutrient-Rich Food Index (NRF) 9.3

The NRF9.3 represents the nutrient density of the total diet with a maximum score of 900 [8; 13]. It includes nine qualifying nutrients (protein, dietary fiber, vitamins A, C, and D, calcium, iron, potassium, and magnesium) and three disqualifying nutrients (added sugars, saturated fats, and sodium). We calculated the NRF9.3 according to a previous study in Japan. The NRF9.3 was calculated by subtracting the sum of the share that exceeded percentages of reference daily values (RDVs) for three disqualifying nutrients from the sum of the percentage of RDVs for nine qualifying nutrients. RDVs were derived from the 2020 Japanese DRIs [14] and the conditional recommendation for added sugar advocated by the World Health Organization (<5% of energy) [15]; they are shown in Supplemental Table 1. The nutrient intake of each participant was energy adjusted using estimated energy requirement (EER): adjusted nutrient intake (unit/day) = observed intake (unit/day) × EER (kcal/day)/observed energy intake (kcal/day). Sex- and age-specific EER values for a moderate level of physical activity were derived from the 2020 Japanese DRIs. For qualifying nutrients, the percentage of RDVs was capped at 100% so that high intake of qualifying nutrients would not compensate for low intake of other qualifying nutrients.

6. Diet quality score for Japanese (DQSJ)

The DQSJ consists of 10 components, each given 0–3 points based on the quartile of intake of each component in the study population. The highest quartile is assigned 3 points for seven components (fruits, vegetables, whole grains, dairy, nuts, legumes, and fish), while the lowest quartile is assigned 3 points for three components (red and processed meat, sugar-sweetened beverages, and sodium) [1]. Scores were calculated using sex-specific quartile intakes of components (g/1,000 kcal) in this population. A large proportion of participants reported that they did not consume whole grain (95.1%) and nuts (51.6%). Therefore, for whole grains and nuts, 0 points were assigned to non-consumers, while 1 to 3 points were assigned to consumers according to tertile intake among consumers. Major food items included in DQSJ components were as follows: fruits did not include fruit juice or jam; vegetables included vegetables, seaweeds, and mushrooms (excluding pickles, starchy vegetables, vegetable seasoning, and vegetable juice); nuts included peanuts and other nuts; fish included oily and lean fish (excluding fish roe, shellfish, octopus and squid, crustaceans, and processed seafood; SSBs included lactic acid bacteria beverages, fruit juice (excluding 100% fruit juice), cocoa, cola, and other sugar-sweetened soft drink, (including sports drinks), tea and coffee with sugar [1].

7. Japanese Food Guide Spinning Top (JFGST)

We calculated the JFGST as in the previous study using the same cohorts [(16)] but excluding total energy intake due to difficulty in assessing energy intake using the FFQ [(17; 18)]. The JFGST included grain dishes (including rice, bread, and noodles), vegetable dishes (including vegetables, mushrooms, potatoes, and seaweed), fish and meat dishes (including meat, fish, eggs, and soybeans), milk (milk and milk products), and fruits (fruits and 100% fruit juice counted as half the weight). The quantity of specific foods constituting a single serving is estimated on the food guide. One serving of a grain dish provides approximately 40 g of carbohydrates, and one serving of vegetable dish contains around 70 g of the main ingredient. A single serving of fish or meat provides about 6 g of protein. A serving of milk provides approximately 100 mg of calcium. A serving of fruits includes around 100 g of the primary ingredient. In this study, the servings of each component were calculated using information on the quantity of each dish included in the FFQ[(16)]. For example, one hamburger steak was counted as two servings of meat dishes and one serving of a vegetable dish. Following the previous study[(16)], servings were calculated with adjustment for overestimation or underestimation of the FFQ compared with a 3-day dietary record.

The suggested number of servings for each food category is determined by sex, age, and two different levels of physical activity. For this study, low physical activity was defined as a metabolic equivalents (METs)-hours per week level of less than 1.5 per week, and approximately 25% of participants fell under this category. For men aged 70 years or more and women aged 70 years or more with low physical activity, recommended numbers of servings are 4–5 sv for grain dishes, 5–6 sv for vegetable dishes, 3–4 sv for fish and meat dishes, 2 sv for milk, and 1 sv for fruits. For men aged 18–69 years with low physical activity and women aged 18–69 years with moderate or high physical activity, the corresponding values are 5–7 sv, 5–6 sv, 3-5 sv, 2 sv, and 2 sv, respectively, whereas for men aged 18–69 years with moderate or high physical activity, the corresponding values were 7–8 sv, 6–7 sv, 4–6 sv, 2–3 sv, and 2–3 sv, respectively. The recommended kilocalories from snacks and alcoholic beverages together is set at 200 kcal/day for all people.

8. 12-item Japanese diet index (JDI12)

The JDI was developed by adding three beneficial components (soybeans and soybean-derived foods, fruit, and mushrooms) to the original 9-itemJDI [(19)]. The JDI12 consists of 12 components, each given 0 or 1 point based on the median intake of each component (g/day) in the study, resulting in a score range of 0-12. Participants gained 1 point if their intakes of 10 components (rice, miso, fish and shellfish, green and yellow

vegetables, seaweed, pickles, green tea, soybeans and soybean-derived foods, fruits, and mushrooms) were above the median. Participants gained 1 point if their intakes of the two less beneficial components (beef and pork, coffee) were below the median. In this study, rice included both white and brown rice. Miso was taken to mean miso soup. Coffee included coffee with or without caffeine. Energy adjustment was not performed for component intake, following the original and previous papers [19; 20]

Supplemental Table 1 Reference daily values used for the calculation of the Nutrient-Rich Food Index 9.3 (NRF9.3) for Japanese adults

	Men				Women			
	30–49 y	50–64 y	65–74 y	75 y or more	30–49 y	50–64 y	65–74 y	75 y or more
Estimated energy requirement (kcal)[1]	2,700	2,600	2,400	2,100	2,050	1,950	1,850	1,650
Qualifying nutrients								
Protein (g)[2]	65	65	60	60	50	50	50	50
Fiber (g)[3]	21	21	20	20	18	18	17	17
Vitamin A (µgRAE)[2]	900	900	850	800	700	700	700	650
Vitamin C (mg)[2]	100	100	100	100	100	100	100	100
Vitamin D (mg)[4]	8.5	8.5	8.5	8.5	8.5	8.5	8.5	8.5
Calcium (mg)[2]	750	750	750	700	650	650	650	600
Iron (mg)[2]	7.5	7.5	7.5	7	10.5	6.5	6	6
Potassium (mg)[3]	3,000	3,000	3,000	3,000	2,600	2,600	2,600	2,600
Magnesium (mg)[2]	370	370	350	320	290	290	280	260
Disqualifying nutrients								
Added sugars (% energy)	10	10	10	10	10	10	10	10
Saturated fats (% energy)[3]	7	7	7	7	7	7	7	7
Sodium (g salt equivalent)[3]	7.5	7.5	7.5	7.5	6.5	6.5	6.5	6.5

RDVs, reference daily values; RAE, retinol activity equivalent. Values were derived from the Dietary Reference Intakes for Japanese, 2020 [(14)], except for added sugar, determined based on the World Health Organization's conditional recommendation [(15)].

[1] Estimated energy requirement for moderate level of physical activity

[2] Recommended dietary allowance

[3] Tentative dietary goal for preventing lifestyle-related disease

[4] Adequate intake

Supplemental Table 2 The mean and standard deviation (SD) of the diet quality indices in the whole and validation sample and spearman correlation coefficients between the diet quality indices derived from the food frequency questionnaire (FFQ) and 12-day dietary record (DR).

	Whole sample (n=29,079)		Validation sample (n=37)				
	FFQ		FFQ		DR		Spearman correlation coefficients
	Mean	SD	Mean	SD	Mean	SD	between FFQ and DR
DASH	21.8	3.8	22.8	5.7	23.1	6.2	0.79
AMED	3.8	1.6	4.2	2.1	4.4	1.9	0.52
HEI-2015	54.7	4.5	57.5	6.0	54.3	6.4	0.59
AHEI-2010	62.3	7.8	67.6	11.2	65.5	7.2	0.74
NRF9.3	724	57	736.3	65.4	700	73	0.68
DQSJ	13.2	3.5	14.1	5.1	14.6	5.7	0.86
JFGST	33.8	8.4	-	-	-	-	-
JDI12	6.0	2.5	6.2	2.9	6.1	2.9	0.72

DASH, Dietary Approaches to Stop Hypertension; AMED, Alternate Mediterranean Diet; HEI-2015, Healthy Eating Index-2015; AHEI-2010, Alternate Healthy Eating Index-2010; NRF9.3, Nutrient Rich Food Score 9.3; DQSJ, Diet Quality Score for Japanese; JFGST, Japanese Food Guide Spinning Top; JDI12, 12-item Japanese Diet Index.

The JFGST was not calculated in the validation sample because the dietary records retained only food items without dish names.

Supplemental Table 3 Hazard ratios of mortality from major cancers for 1 SD differences in the 8 diet quality indices among Japanese adults (n=29,079)

	Trachea, bronchus, and lung cancer (n of death = 296)		Stomach cancer (n of death = 293)		Colorectal cancer (n of death = 225)		Liver cancer (n of death = 138)		Pancreatic cancer (n of death = 120)	
DASH	0.96	(0.85 to 1.08)	1.05	(0.93 to 1.19)	1.01	(0.88 to 1.16)	1.11	(0.93 to 1.32)	1.07	(0.88 to 1.29)
AMED	0.93	(0.83 to 1.05)	1.02	(0.91 to 1.15)	0.95	(0.83 to 1.09)	0.84	(0.70 to 1.00)	0.96	(0.79 to 1.15)
HEI-2015	0.93	(0.82 to 1.04)	1.05	(0.93 to 1.18)	1.04	(0.91 to 1.19)	0.94	(0.79 to 1.11)	0.85	(0.71 to 1.03)
AHEI-2010	0.91	(0.80 to 1.03)	1.00	(0.88 to 1.13)	0.98	(0.85 to 1.13)	0.93	(0.78 to 1.11)	1.14	(0.94 to 1.38)
NRF9.3	0.91	(0.82 to 1.01)	0.98	(0.88 to 1.10)	0.93	(0.82 to 1.05)	1.01	(0.85 to 1.19)	0.86	(0.73 to 1.01)
DQSJ	0.89	(0.79 to 1.01)	0.97	(0.85 to 1.09)	1.03	(0.89 to 1.18)	1.11	(0.93 to 1.32)	1.14	(0.94 to 1.38)
JFGST	0.99	(0.87 to 1.13)	0.96	(0.84 to 1.09)	0.93	(0.80 to 1.08)	1.22	(1.01 to 1.48)	1.03	(0.84 to 1.26)
JDI12	0.91	(0.79 to 1.05)	1.07	(0.93 to 1.23)	0.94	(0.81 to 1.11)	0.95	(0.78 to 1.17)	1.06	(0.85 to 1.31)

SD, standard deviation; DASH, Dietary Approaches to Stop Hypertension; AMED, Alternate Mediterranean Diet; HEI-2015, Healthy Eating Index-2015; AHEI-2010, Alternate Healthy Eating Index-2010; NRF9.3, Nutrient Rich Food Score 9.3; DQSJ, Diet Quality Score for Japanese; JFGST, Japanese Food Guide Spinning Top; JDI12, 12-item Japanese Diet Index.

The values of SD for each score are presented in Supplemental Table 2.

The models were adjusted for age, sex, total energy intake (continuous), body mass index (continuous, as quadratic term), physical activity (continuous), smoking (packs per year, continuous), education (less than 12 years, 12–14 years, 15 years or more), marital status, sleep duration (continuous, as quadratic term), history of hypertension (yes or no), history of diabetes (yes or no), multivitamin use (yes or no), menopause status (yes or no, only for women).

Supplemental Table 4 Hazard ratios (HRs) (95% CIs) for mortality from all causes by quartiles of each diet quality indices among Japanese adults (n=29,079)

Diet quality		N of	N of	Model 1			Model 2		
indices	Mean score (range)	participants	death	HR	95%CI	p trend	HR	95%CI	p trend
DASH									
Q1	16.9 (9–19)	7,032	930	1.00	ref	<0.001	1.00	ref	0.002
Q2	20.3 (19–21)	6,947	1,261	0.92	(0.84 to 0.997)		0.91	(0.83 to 0.99)	
Q3	22.9 (22–24)	8,262	1,652	0.86	(0.80 to 0.94)		0.88	(0.81 to 0.95)	
Q4	26.9 (25–37)	6,838	1,496	0.84	(0.78 to 0.92)		0.87	(0.80 to 0.95)	
AMED									
Q1	1.6 (0–2)	6,286	1,148	1.00	ref	<0.001	1.00	ref	<0.001
Q2	3.0 (3–3)	6,195	1,140	0.92	(0.85 to 1.001)		0.96	(0.88 to 1.04)	
Q3	4.0 (4–4)	6,806	1,265	0.84	(0.78 to 0.91)		0.89	(0.82 to 0.97)	
Q4	5.6 (5–9)	9,792	1,786	0.81	(0.75 to 0.87)		0.88	(0.81 to 0.95)	
HEI-2015									
Q1	49 (31–52)	7,269	1,428	1.00	ref	<0.001	1.00	ref	0.061
Q2	53 (51–55)	7,270	1,313	0.93	(0.87 to 1.01)		0.95	(0.88 to 1.02)	
Q3	56 (54–58)	7,270	1,261	0.89	(0.83 to 0.96)		0.94	(0.87 to 1.01)	
Q4	60 (57–76)	7,270	1,337	0.86	(0.80 to 0.93)		0.93	(0.86 to 1.004)	
AHEI-2010									
Q1	53 (31–59)	7,269	1,022	1.00	ref	<0.001	1.00	ref	<0.001
Q2	60 (55–64)	7,270	1,322	0.93	(0.86 to 1.01)		0.93	(0.85 to 1.01)	
Q3	65 (61–68)	7,270	1,467	0.88	(0.81 to 0.95)		0.88	(0.81 to 0.95)	
Q4	72 (66–96)	7,270	1,528	0.77	(0.71 to 0.83)		0.78	(0.72 to 0.85)	

(continued)

(Supplemental Table 4, continued)

Diet quality indices	Mean score (range)	N of participants	N of death	Model 1 HR	95%CI	p trend	Model 2 HR	95%CI	p trend
NRF9.3									
Q1	648 (163–701)	7,269	1,469	1.00	ref	<0.001	1.00	ref	<0.001
Q2	717 (694–735)	7,270	1,217	0.85	(0.79 to 0.92)		0.89	(0.83 to 0.96)	
Q3	748 (732–763)	7,270	1,212	0.81	(0.75 to 0.87)		0.84	(0.78 to 0.91)	
Q4	783 (762–878)	7,270	1,441	0.79	(0.74 to 0.85)		0.84	(0.78 to 0.91)	
DQSJ									
Q1	8.6 (0–10)	6,334	900	1.00	ref	<0.001	1.00	ref	<0.001
Q2	12.1 (11–13)	9,149	1,614	0.91	(0.79 to 1.05)		0.89	(0.82 to 0.96)	
Q3	14.5 (14–15)	6,052	1,192	0.78	(0.67 to 0.92)		0.86	(0.79 to 0.94)	
Q4	17.6 (16–26)	7,544	1,633	0.75	(0.65 to 0.87)		0.82	(0.75 to 0.89)	
JFGST									
Q1	23 (0–30)	7,269	1,350	1.00	ref	0.01	1.00	ref	<0.001
Q2	31 (26–36)	7,270	1,268	0.94	(0.87 to 1.01)		0.89	(0.82 to 0.96)	
Q3	37 (32–41)	7,270	1,298	0.88	(0.82 to 0.95)		0.84	(0.77 to 0.91)	
Q4	44 (37–58)	7,270	1,423	0.92	(0.85 to 0.99)		0.87	(0.80 to 0.94)	
JDI12									
Q1	3.0 (0–4)	8,782	1,504	1.00	ref	<0.001	1.00	ref	0.002
Q2	5.5 (5–6)	7,457	1,333	0.84	(0.78 to 0.90)		0.87	(0.80 to 0.93)	
Q3	7.3 (7–8)	5,502	1,129	0.84	(0.77 to 0.90)		0.89	(0.82 to 0.96)	
Q4	9.2 (8–12)	7,338	1,373	0.79	(0.73 to 0.85)		0.87	(0.80 to 0.95)	

DASH, Dietary Approaches to Stop Hypertension; AMED, Alternate Mediterranean Diet; HEI-2015, Healthy Eating Index-2015; AHEI-2010, Alternate Healthy Eating Index-2010; NRF9.3, Nutrient Rich Food Score 9.3; DQSJ, Diet Quality Score for Japanese; JFGST, Japanese Food Guide Spinning Top; JDI12, 12-item Japanese Diet Index.

Model 1, adjusted for age and sex

Model 2, adjusted for age, sex, total energy intake (continuous), body mass index (continuous, as quadratic term), physical activity (continuous), smoking (pack year, continuous), education (less than 12 years, 12-14 years, 15 years or more), marital status, sleep duration (continuous, as quadratic term), history of hypertension (yes or no), history of diabetes (yes or no), multivitamin use (yes or no), menopause status (yes or no, only for women).

P for trend across increasing quartile of diet quality indices by assigning the mean value to each category and treating them as continuous.

The participants were first categorized into four groups based on sex-specific quartiles of the indices and then analyzed together, resulting in overlapping score ranges.

Supplemental Table 5 Hazard ratios (HRs) (95% CIs) for mortality from cardiovascular diseases (CVD) and cancer by quartiles of each diet quality indices among Japanese adults (n=29,079)

			CVD				Cancer			
Diet quality indices	Mean score (range)	N of participants	N of death	HR	95% CI	p for trend	N of death	HR	95% CI	p for trend
DASH	21.8 (9–37)									
Q1	16.9 (9–19)	7,032	296	1.00	ref	<0.001	278	1.00	ref	0.10
Q2	20.3 (19–21)	6,947	411	0.92	(0.79 to 1.07)		362	0.95	(0.81 to 1.11)	
Q3	22.9 (22–24)	8,262	516	0.81	(0.70 to 0.94)		513	1.05	(0.90 to 1.22)	
Q4	26.9 (25–37)	6,838	455	0.77	(0.67 to 0.90)		467	1.09	(0.94 to 1.27)	
AMED	3.8 (0–9)									
Q1	1.6 (0–2)	6,286	348	1.00	ref	0.08	325	1.00	ref	0.97
Q2	3.0 (3–3)	6,195	386	1.08	(0.94 to 1.25)		334	0.98	(0.84 to 1.15)	
Q3	4.0 (4–4)	6,806	398	0.94	(0.81 to 1.09)		373	0.94	(0.81 to 1.09)	
Q4	5.6 (5–9)	9,792	546	0.92	(0.80 to 1.06)		588	1.00	(0.87 to 1.15)	
HEI-2015	55 (31–76)									
Q1	49 (31–52)	7,269	477	1.00	ref	0.11	395	1.00	ref	0.89
Q2	53 (51–55)	7,270	402	0.89	(0.78 to 1.01)		407	1.04	(0.90 to 1.19)	
Q3	56 (54–58)	7,270	394	0.92	(0.81 to 1.05)		396	1.02	(0.88 to 1.17)	
Q4	60 (57–76)	7,270	405	0.89	(0.77 to 1.02)		422	1.02	(0.88 to 1.17)	
AHEI-2010	62 (31–96)									
Q1	53 (31–59)	7,269	323	1.00	ref	<0.001	292	1.00	ref	0.91
Q2	60 (55–64)	7,270	422	0.92	(0.79 to 1.06)		393	1.04	(0.89 to 1.21)	
Q3	65 (61–68)	7,270	433	0.80	(0.69 to 0.92)		468	1.11	(0.95 to 1.28)	
Q4	72 (66–96)	7,270	500	0.77	(0.67 to 0.89)		467	0.99	(0.85 to 1.16)	

(continued)

(Supplemental Table 5, continued)

Diet quality indices	Mean score (range)	N of participants	CVD N of death	CVD HR	CVD 95%CI	CVD p for trend	Cancer N of death	Cancer HR	Cancer 95%CI	Cancer p for trend
NRF9.3	724 (163–878)									
Q1	648 (163–701)	7,269	454	1.00	ref	0.006	442	1.00	ref	0.054
Q2	717 (694–735)	7,270	404	1.00	(0.87 to 1.14)		343	0.79	(0.68 to 0.91)	
Q3	748 (732–763)	7,270	369	0.86	(0.75 to 0.98)		384	0.85	(0.74 to 0.97)	
Q4	783 (762–878)	7,270	451	0.85	(0.75 to 0.97)		451	0.89	(0.78 to 1.01)	
DQSJ	13.2 (0–26)									
Q1	8.6 (0–10)	6,334	278	1.00	ref	<0.001	282	1.00	ref	0.31
Q2	12.1 (11–13)	9,149	535	0.91	(0.79 to 1.05)		439	0.84	(0.72 to 0.98)	
Q3	14.5 (14–15)	6,052	358	0.80	(0.68 to 0.93)		359	0.92	(0.79 to 1.08)	
Q4	17.6 (16–26)	7,544	507	0.78	(0.67 to 0.90)		540	1.01	(0.87 to 1.17)	
JFGST	34 (0–58)									
Q1	23 (0–30)	7,269	414	1.00	ref	0.01	413	1.00	ref	0.47
Q2	31 (26–36)	7,270	407	0.89	(0.77 to 1.02)		379	0.92	(0.79 to 1.06)	
Q3	37 (32–41)	7,270	402	0.80	(0.69 to 0.92)		396	0.91	(0.78 to 1.05)	
Q4	44 (37–58)	7,270	455	0.85	(0.73 to 0.98)		432	0.95	(0.82 to 1.10)	
JDI12	6.0 (0–12)									
Q1	3.0 (0–4)	8,782	497	1.00	ref	0.007	409	1.00	ref	0.30
Q2	5.5 (5–6)	7,457	427	0.83	(0.72 to 0.94)		397	0.98	(0.85 to 1.13)	
Q3	7.3 (7–8)	5,502	326	0.82	(0.71 to 0.95)		374	1.07	(0.93 to 1.25)	
Q4	9.2 (8–12)	7,338	428	0.82	(0.7 to 0.95)		440	1.07	(0.91 to 1.25)	

DASH, Dietary Approaches to Stop Hypertension; AMED, Alternate Mediterranean Diet; HEI-2015, Healthy Eating Index-2015; AHEI-2010, Alternate Healthy Eating Index-2010; NRF9.3, Nutrient Rich Food Score 9.3; DQSJ, Diet Quality Score for Japanese; JFGST, Japanese Food Guide Spinning Top; JDI12, 12-item Japanese Diet Index.

Adjusted for age, sex, total energy intake (continuous), body mass index (continuous, as quadratic term), physical activity (continuous), smoking (pack year, continuous), education (less than 12 years, 12-14 years, 15 years or more), marital status, sleep duration (continuous, as quadratic term), history of hypertension (yes or no), history of diabetes (yes or no), multivitamin use (yes or no), menopause status (yes or no, only for women).

P for trend across increasing quartile of diet quality indices by assigning the mean value to each category and treating them as continuous.

The participants were first categorized into four groups based on sex-specific quartiles of the indices and then analyzed together, resulting in overlapping score ranges.

Supplemental Table 6 Hazard ratios of mortality of all-cause, CVD, cancer, and other causes for 1 SD increments in the 8 diet quality indices among Japanese adults with various assumptions

	Excluding death occurring during the first three years n=28,536		Those without multivitamin use n=18,731		Adding alcohol intake as a covariate n=29,079		Maximum follow–up period was set 8 years n=29,079		Complete death analysis n=23,463	
	HR	95% CI	HR	95% CI	HR	95% CI	HR	95% CI	HR	95% CI
All causes	n of death = 4,616		n of death = 3,088		n of death = 5,339		n of death = 2,662		n of death = 3,651	
DASH	0.95	(0.92 to 0.98)	0.94	(0.91 to 0.98)	0.94	(0.91 to 0.966)	0.93	(0.89-0.97)	0.94	(0.91 to 0.98)
AMED	0.94	(0.91 to 0.97)	0.95	(0.91 to 0.98)	0.94	(0.91 to 0.97)	0.92	(0.89-0.96)	0.94	(0.91 to 0.97)
HEI-2015	0.96	(0.93 to 0.99)	0.96	(0.92 to 0.99)	0.97	(0.94 to 0.99)	0.94	(0.91-0.98)	0.95	(0.92 to 0.98)
AHEI-2010	0.91	(0.88 to 0.94)	0.89	(0.85 to 0.92)	0.90	(0.87 to 0.93)	0.88	(0.84-0.92)	0.89	(0.86 to 0.92)
NRF9.3	0.93	(0.91 to 0.96)	0.94	(0.91 to 0.97)	0.93	(0.90 to 0.95)	0.91	(0.88-0.94)	0.93	(0.91 to 0.96)
DQSJ	0.93	(0.90 to 0.96)	0.91	(0.88 to 0.95)	0.92	(0.89 to 0.94)	0.91	(0.87-0.95)	0.91	(0.88 to 0.94)
JFGST	0.95	(0.92 to 0.98)	0.94	(0.91 to 0.98)	0.93	(0.90 to 0.96)	0.95	(0.91-0.998)	0.94	(0.91 to 0.98)
JDI12	0.95	(0.91 to 0.98)	0.94	(0.90 to 0.98)	0.94	(0.91 to 0.97)	0.91	(0.87-0.95)	0.95	(0.91 to 0.98)
CVD	n of death = 1,429		n of death = 959		n of death = 1,678		n of death = 838		n of death = 1,095	
DASH	0.92	(0.87 to 0.97)	0.93	(0.87 to 1.00)	0.90	(0.85 to 0.95)	0.88	(0.82-0.95)	0.90	(0.84 to 0.96)
AMED	0.95	(0.90 to 1.01)	0.95	(0.89 to 1.02)	0.95	(0.90 to 0.995)	0.93	(0.87-1.003)	0.94	(0.88 to 0.998
HEI-2015	0.98	(0.93 to 1.03)	0.98	(0.91 to 1.04)	0.98	(0.93 to 1.03)	0.94	(0.88-1.01)	0.96	(0.9 to 1.02)
AHEI-2010	0.91	(0.86 to 0.96)	0.88	(0.81 to 0.94)	0.89	(0.84 to 0.93)	0.85	(0.79-0.92)	0.87	(0.82 to 0.93)
NRF9.3	0.93	(0.89 to 0.98)	0.94	(0.89 to 0.99)	0.93	(0.89 to 0.97)	0.91	(0.86-0.96)	0.93	(0.88 to 0.98)
DQSJ	0.91	(0.86 to 0.97)	0.91	(0.85 to 0.97)	0.89	(0.84 to 0.93)	0.87	(0.81-0.94)	0.88	(0.83 to 0.94)
JFGST	0.94	(0.89 to 0.9998)	0.92	(0.86 to 0.99)	0.91	(0.86 to 0.97)	0.93	(0.86-1.01)	0.94	(0.88 to 1.01)
JDI12	0.93	(0.87 to 0.99)	0.92	(0.85 to 0.997)	0.91	(0.86 to 0.97)	0.89	(0.82-0.97)	0.9	(0.84 to 0.97)
Cancer	n of death = 1,401		n of death = 956		n of death = 1,620		n of death = 848		n of death = 1,199	
DASH	1.03	(0.97 to 1.09)	1.02	(0.95 to 1.09)	1.04	(0.98 to 1.09)	1.02	(0.95-1.09)	1.04	(0.98 to 1.1)
AMED	0.98	(0.92 to 1.03)	1.01	(0.95 to 1.08)	0.99	(0.94 to 1.04)	0.98	(0.91-1.05)	0.96	(0.91 to 1.02)
HEI-2015	0.97	(0.92 to 1.02)	0.97	(0.91 to 1.04)	0.98	(0.93 to 1.03)	0.96	(0.89-1.03)	0.96	(0.91 to 1.02)
AHEI-2010	1.00	(0.94 to 1.06)	0.98	(0.92 to 1.05)	0.99	(0.94 to 1.05)	0.98	(0.91-1.06)	1.00	(0.94 to 1.06)
NRF9.3	0.95	(0.91 to 1.005)	0.97	(0.92 to 1.03)	0.95	(0.91 to 1.001)	0.94	(0.89-1.003)	0.95	(0.90 to 0.999
DQSJ	1.01	(0.95 to 1.06)	0.99	(0.93 to 1.06)	1.01	(0.96 to 1.07)	1.03	(0.95-1.1)	1.00	(0.95 to 1.06)
JFGST	0.99	(0.93 to 1.05)	0.97	(0.91 to 1.04)	0.99	(0.94 to 1.05)	0.99	(0.92-1.07)	0.96	(0.9 to 1.02)
JDI12	1.01	(0.95 to 1.08)	1.00	(0.92 to 1.08)	1.02	(0.96 to 1.08)	1.01	(0.93-1.1)	1.02	(0.96 to 1.10)

DASH, Dietary Approaches to Stop Hypertension; AMED, Alternate Mediterranean Diet; HEI-2015, Healthy Eating Index-2015; AHEI-2010, Alternate Healthy Eating Index-2010; NRF9.3, Nutrient Rich Food Score 9.3; DQSJ, Diet Quality Score for Japanese; JFGST, Japanese Food Guide Spinning Top; JDI12, 12-item Japanese Diet Index.

Adjusted for age, sex, total energy intake (continuous), body mass index (continuous, as quadratic term), physical activity (continuous), smoking (pack year, continuous), education (less than 12 years, 12-14 years, 15 years or more), marital status, sleep duration (continuous, as quadratic term), history of hypertension (yes or no), history of diabetes (yes or no), multivitamin use (yes or no), menopause status (yes or no, only for women)

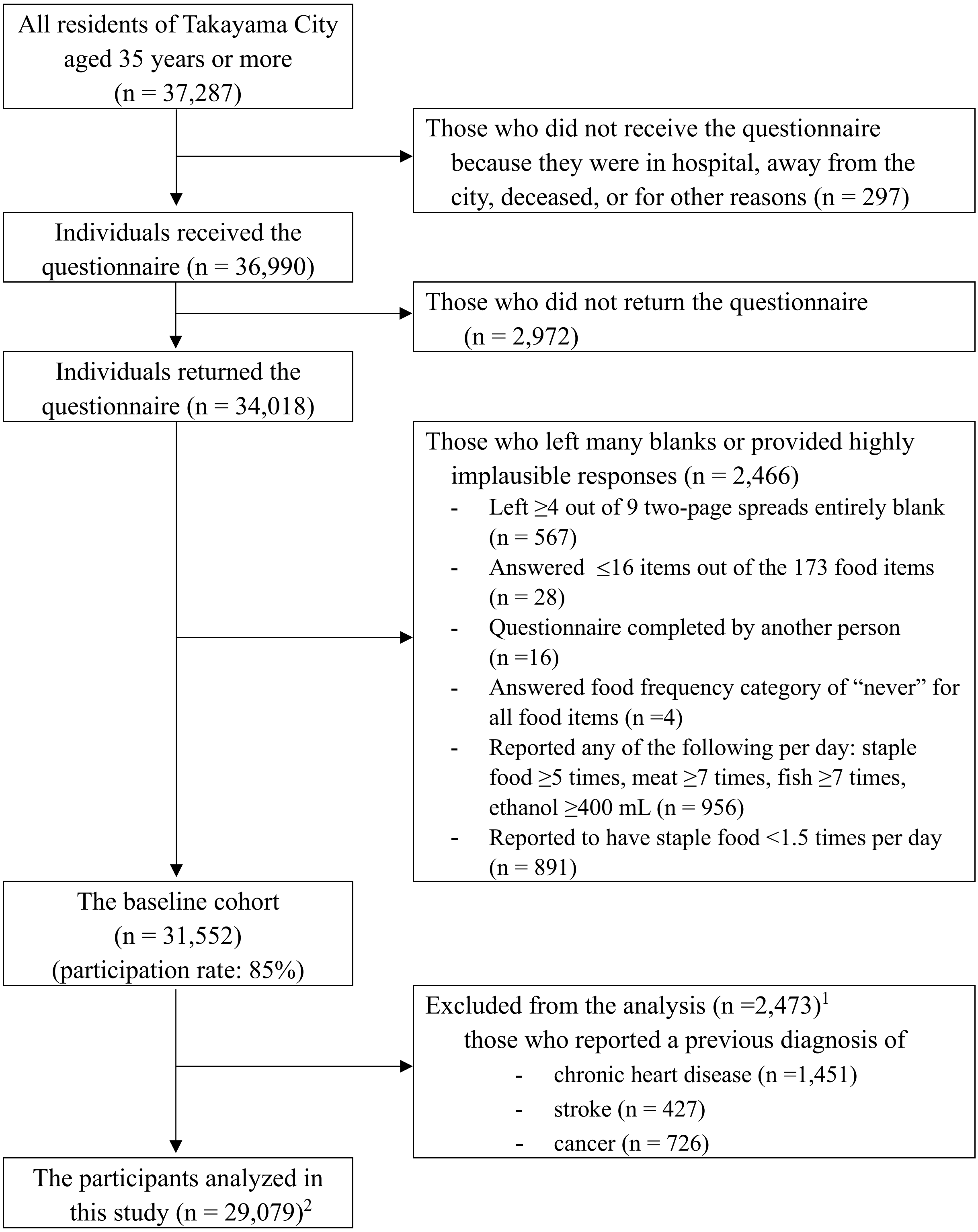

Supplemental Figure 1 Flowchart of participant selection in the Takayama study.

[1] The sum of the three categories exceeds the total number excluded because some participants reported more than one condition.

[2] Due to the exclusion criteria, dietary data from the FFQ was available for all participants.

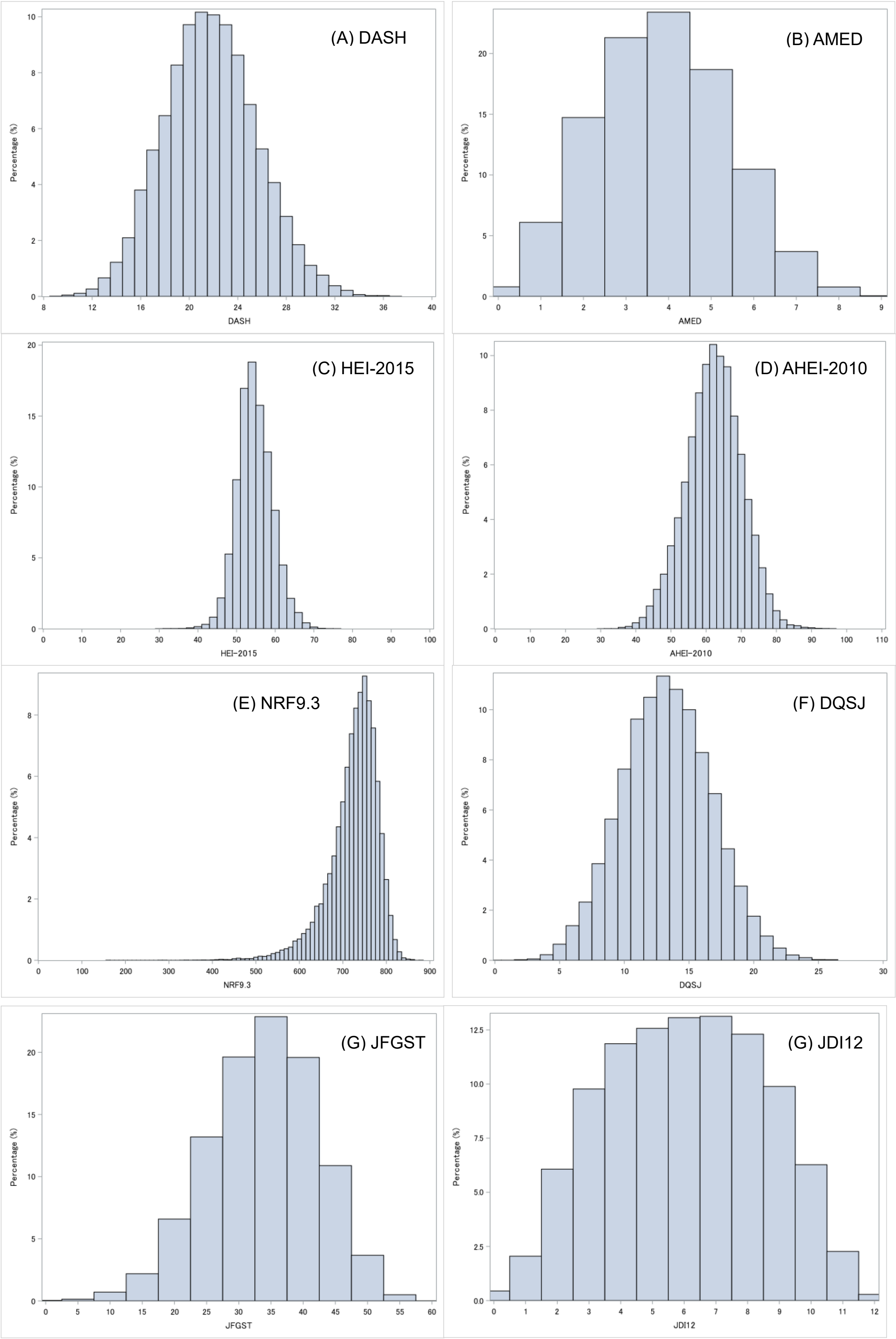
(A) DASH
Percentage (%)
DASH
(B) AMED
Percentage (%)
AMED
(C) HEI-2015
Percentage (%)
HEI−2015
(D) AHEI-2010
Percentage (%)
AHEI−2010
(E) NRF9.3
Percentage (%)
NRF9.3
(F) DQSJ
Percentage (%)
DQSJ
(G) JFGST
Percentage (%)
JFGST
(G) JDI12
Percentage (%)
JDI12

Supplemental Figure 2 Distribution of 8 diet quality indices in the Takayama study.
(A) Dietary Approaches to Stop Hypertension (DASH); (B) Alternate Mediterranean Diet (AMED); (C) Healthy Eating Index-2015 (HEI-2015); (D) Alternate Healthy Eating Index-2010 (AHEI-2010); (E) Nutrient Rich Food Score 9.3 (NRF9.3); (F) Diet Quality Score for Japanese (DQSJ); (G) Japanese Food Guide Spinning Top (JFGST); (H) 12-item Japanese Diet Index (JDI12).

	DASH	AMED	HEI	AHEI	NRF	DQSJ	JFGST	JDI12
Fiber	0.48	0.53	0.52	0.53	0.38	0.44	0.34	0.50
Magnesium	0.47	0.54	0.49	0.55	0.36	0.49	0.26	0.53
Iron	0.36	0.52	0.41	0.55	0.28	0.35	0.26	0.51
Total fruits	0.42	0.42	0.65	0.23	0.36	0.41	0.20	0.44
Raw fruits	0.42	0.42	0.65	0.23	0.36	0.41	0.20	0.44
Calcium	0.44	0.24	0.34	0.38	0.35	0.51	0.34	0.34
Non-starchy vegetables	0.32	0.49	0.42	0.25	0.35	0.35	−0.01	0.69
Total vegetables	0.32	0.50	0.43	0.25	0.35	0.35	−0.02	0.69
Vitamin C	0.29	0.44	0.54	0.23	0.36	0.28	0.25	0.40
Legume	0.37	0.48	0.32	0.37	0.21	0.39	−0.10	0.67
Protein	0.20	0.43	0.37	0.37	0.33	0.37	0.15	0.36
Seaweed	0.27	0.38	0.29	0.27	0.24	0.29	0.01	0.7
n-3 fatty acid	0.13	0.54	0.42	0.33	0.30	0.32	0.02	0.41
PUFA	0.14	0.49	0.39	0.39	0.18	0.23	0.11	0.35
Sodium	0.16	0.47	0.32	0.35	0.09	0.21	0.16	0.46
Mushroom	0.15	0.36	0.31	0.14	0.25	0.20	−0.04	0.60
Vitamin D	0.06	0.38	0.23	0.16	0.42	0.33	−0.04	0.30
Dairy (total)	0.30	−0.03	0.18	0.09	0.31	0.41	0.23	0.20
Low-fat dairy	0.40	0.13	0.28	0.04	0.20	0.20	0.11	0.16
Fat	0.04	0.26	0.30	0.21	0.18	0.20	0.11	0.19
Fish	−0.01	0.37	0.22	−0.03	0.33	0.26	−0.31	0.48
Fish and other seafoods	−0.01	0.37	0.22	−0.03	0.33	0.26	−0.31	0.48
Miso	0.12	0.19	0.06	0.19	0.03	0.11	0.00	0.52
Saturated fatty acids	0.08	0.05	0.15	0.11	0.22	0.24	0.19	0.09
Green tea	0.10	0.11	0.14	0.12	0.17	0.11	0.11	0.27
Whole grain	0.26	0.29	0.12	0.06	0.05	0.25	−0.02	0.08
Nuts	0.02	0.36	0.14	−0.01	0.10	0.27	−0.08	0.12
MUFA	−0.10	0.24	0.27	0.09	0.13	0.06	0.03	0.12
Pickled vegetables	0.01	0.19	0.13	−0.01	−0.01	0.03	−0.09	0.45
Carbohydrate	0.07	−0.16	−0.19	0.03	−0.06	−0.12	0.26	−0.15
Rice	−0.08	−0.06	−0.19	−0.17	−0.01	−0.11	−0.26	0.29
Coffee	−0.17	−0.03	−0.05	−0.17	−0.03	−0.14	−0.08	−0.21
Red and processed meat	−0.35	0.04	0.09	−0.29	0.01	−0.23	−0.29	0.15
Alcohol intake	−0.15	0.00	−0.07	−0.18	−0.07	−0.08	−0.40	0.03
SSBs	−0.32	0.03	0.05	−0.50	0.02	−0.30	−0.19	0.09

Supplemental Figure 3 The Spearman correlation coefficients between the 8 diet quality indices and intakes of selected nutrients and food groups (unit per 1000 kcal).
Green shades indicate positive correlations, and orange shades indicate negative correlations. Darker colors indicate stronger correlations.
DASH, Dietary Approaches to Stop Hypertension; AMED, Alternate Mediterranean Diet; HEI-2015, Healthy Eating Index-2015; AHEI-2010, Alternate Healthy Eating Index-2010; NRF9.3, Nutrient Rich Food Score 9.3; DQSJ, Diet Quality Score for Japanese; JFGST, Japanese Food Guide Spinning Top; JDI12, 12-item Japanese Diet Index; %E, percentage of total energy intake; PUFA, polyunsaturated fatty acids; MUFA, monounsaturated fatty acids; SSBs, sugar-sweetened beverages.

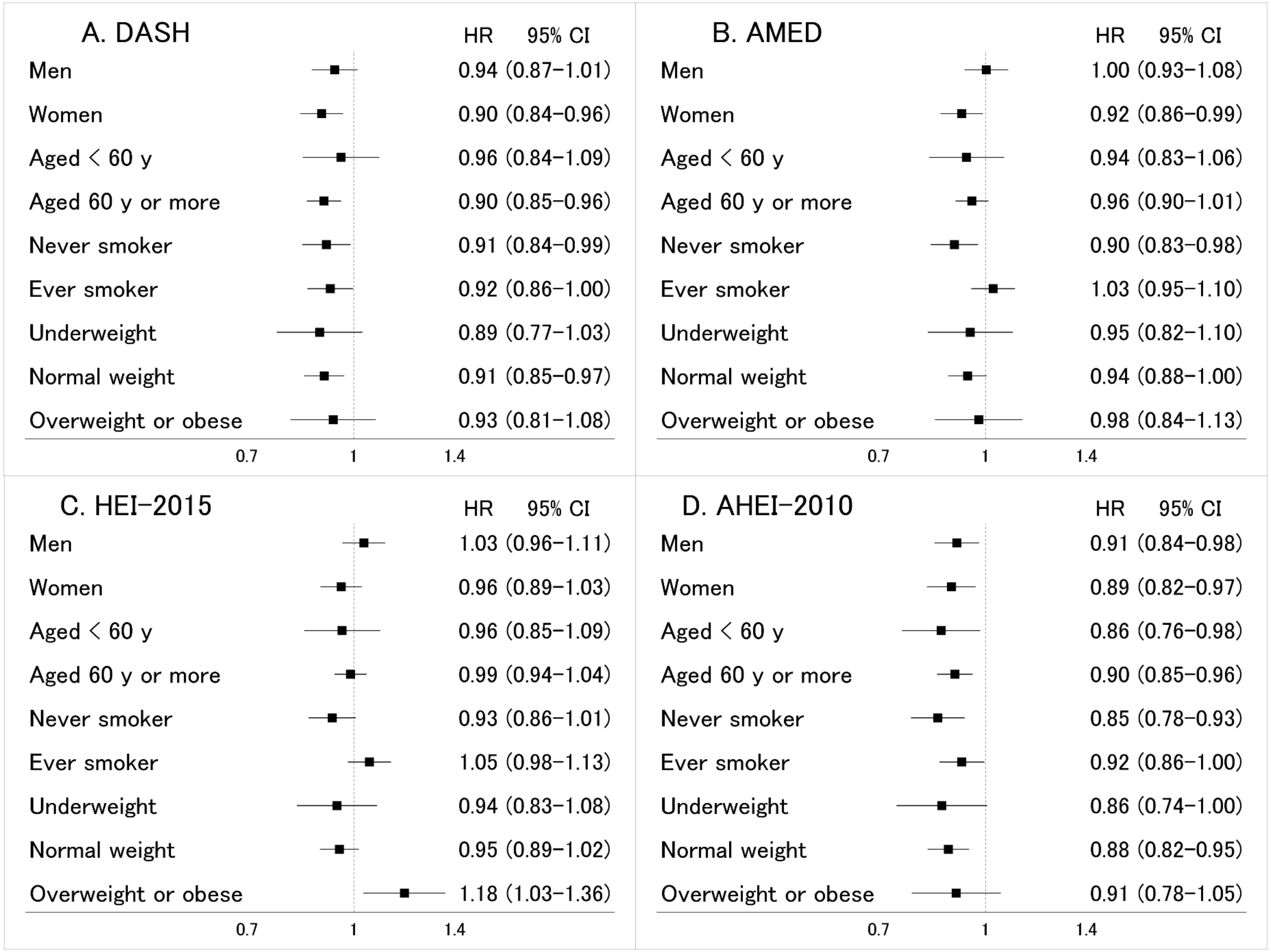
A. DASH
HR 95% CI
Men 0.94 (0.87–1.01)
Women 0.90 (0.84–0.96)
Aged < 60 y 0.96 (0.84–1.09)
Aged 60 y or more 0.90 (0.85–0.96)
Never smoker 0.91 (0.84–0.99)
Ever smoker 0.92 (0.86–1.00)
Underweight 0.89 (0.77–1.03)
Normal weight 0.91 (0.85–0.97)
Overweight or obese 0.93 (0.81–1.08)
0.7 1 1.4
B. AMED
HR 95% CI
Men 1.00 (0.93–1.08)
Women 0.92 (0.86–0.99)
Aged < 60 y 0.94 (0.83–1.06)
Aged 60 y or more 0.96 (0.90–1.01)
Never smoker 0.90 (0.83–0.98)
Ever smoker 1.03 (0.95–1.10)
Underweight 0.95 (0.82–1.10)
Normal weight 0.94 (0.88–1.00)
Overweight or obese 0.98 (0.84–1.13)
0.7 1 1.4
C. HEI–2015
HR 95% CI
Men 1.03 (0.96–1.11)
Women 0.96 (0.89–1.03)
Aged < 60 y 0.96 (0.85–1.09)
Aged 60 y or more 0.99 (0.94–1.04)
Never smoker 0.93 (0.86–1.01)
Ever smoker 1.05 (0.98–1.13)
Underweight 0.94 (0.83–1.08)
Normal weight 0.95 (0.89–1.02)
Overweight or obese 1.18 (1.03–1.36)
0.7 1 1.4
D. AHEI–2010
HR 95% CI
Men 0.91 (0.84–0.98)
Women 0.89 (0.82–0.97)
Aged < 60 y 0.86 (0.76–0.98)
Aged 60 y or more 0.90 (0.85–0.96)
Never smoker 0.85 (0.78–0.93)
Ever smoker 0.92 (0.86–1.00)
Underweight 0.86 (0.74–1.00)
Normal weight 0.88 (0.82–0.95)
Overweight or obese 0.91 (0.78–1.05)
0.7 1 1.4

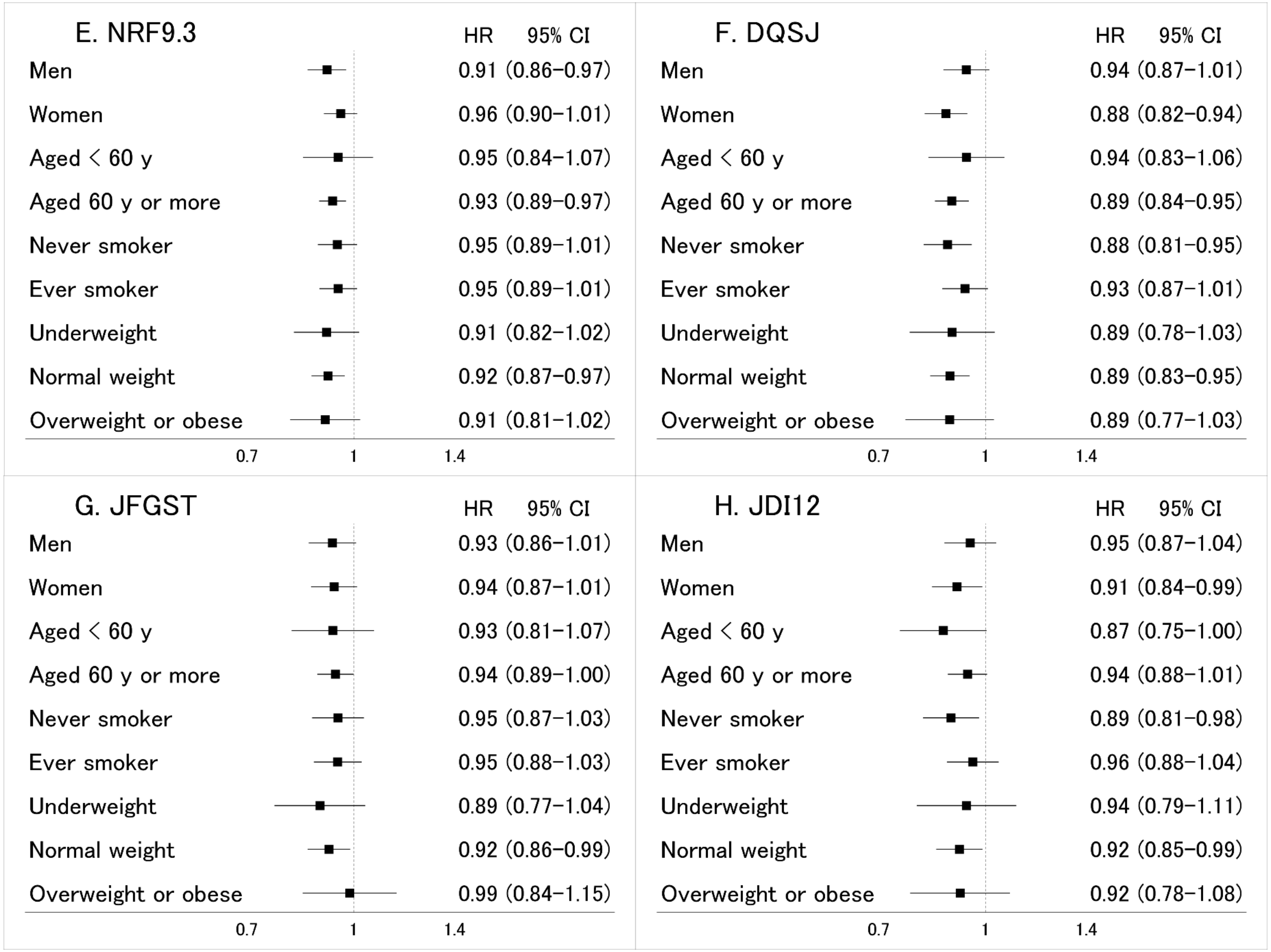
E. NRF9.3
HR 95% CI
Men 0.91 (0.86–0.97)
Women 0.96 (0.90–1.01)
Aged < 60 y 0.95 (0.84–1.07)
Aged 60 y or more 0.93 (0.89–0.97)
Never smoker 0.95 (0.89–1.01)
Ever smoker 0.95 (0.89–1.01)
Underweight 0.91 (0.82–1.02)
Normal weight 0.92 (0.87–0.97)
Overweight or obese 0.91 (0.81–1.02)
0.7 1 1.4
F. DQSJ
HR 95% CI
Men 0.94 (0.87–1.01)
Women 0.88 (0.82–0.94)
Aged < 60 y 0.94 (0.83–1.06)
Aged 60 y or more 0.89 (0.84–0.95)
Never smoker 0.88 (0.81–0.95)
Ever smoker 0.93 (0.87–1.01)
Underweight 0.89 (0.78–1.03)
Normal weight 0.89 (0.83–0.95)
Overweight or obese 0.89 (0.77–1.03)
0.7 1 1.4
G. JFGST
HR 95% CI
Men 0.93 (0.86–1.01)
Women 0.94 (0.87–1.01)
Aged < 60 y 0.93 (0.81–1.07)
Aged 60 y or more 0.94 (0.89–1.00)
Never smoker 0.95 (0.87–1.03)
Ever smoker 0.95 (0.88–1.03)
Underweight 0.89 (0.77–1.04)
Normal weight 0.92 (0.86–0.99)
Overweight or obese 0.99 (0.84–1.15)
0.7 1 1.4
H. JDI12
HR 95% CI
Men 0.95 (0.87–1.04)
Women 0.91 (0.84–0.99)
Aged < 60 y 0.87 (0.75–1.00)
Aged 60 y or more 0.94 (0.88–1.01)
Never smoker 0.89 (0.81–0.98)
Ever smoker 0.96 (0.88–1.04)
Underweight 0.94 (0.79–1.11)
Normal weight 0.92 (0.85–0.99)
Overweight or obese 0.92 (0.78–1.08)
0.7 1 1.4

Supplemental Figure 4 Hazard ratios (HR) on cardiovascular disease mortality per 1 SD increment of each diet quality indices in the Takayama study, stratified by basic characteristics (sex, age, smoking status, and weight status) [a]

DASH, Dietary Approaches to Stop Hypertension; AMED, Alternate Mediterranean Diet; HEI-2015, Healthy Eating Index-2015; AHEI-2010, Alternate Healthy Eating Index-2010; NRF9.3, Nutrient Rich Food Score 9.3; DQSJ, Diet Quality Score for Japanese; JFGST, Japanese Food Guide Spinning Top; JDI12, 12-item Japanese Diet Index.

[a] The horizontal axis for HR is scaled logarithmically. Cox proportional hazards models to estimate HRs and 95% CIs were adjusted for age, sex, total energy intake (continuous), body mass index (continuous, as quadratic term), physical activity (continuous), smoking (pack year, continuous), education (less than 12 years, 12–14 years, 15 years or more), marital status, sleep duration (continuous, as quadratic term), history of hypertension (yes or no), history of diabetes (yes or no), multivitamin use (yes or no), menopause status (yes or no, only for women). Participant numbers and cases were 13,355 and 755 for men, 15,724 and 903 for women, 19,128 and 261 for participants aged less than 60 y, 9,951 and 1,417 for participants aged 60 y or more, 13,802 and 696 for never smokers, 13,257 and 775 for ever smokers, 3,895 and 468 for underweight, 21,101 and 996 for normal weight, and 4,083 and 214 for overweight.

Significant interactions on CVD mortality were observed between the HEI-2015 and smoking status ($p = 0.02$) and BMI ($p = 0.01$), between the JFGST and age ($p = 0.02$), and between AMED and smoking status ($p = 0.04$).

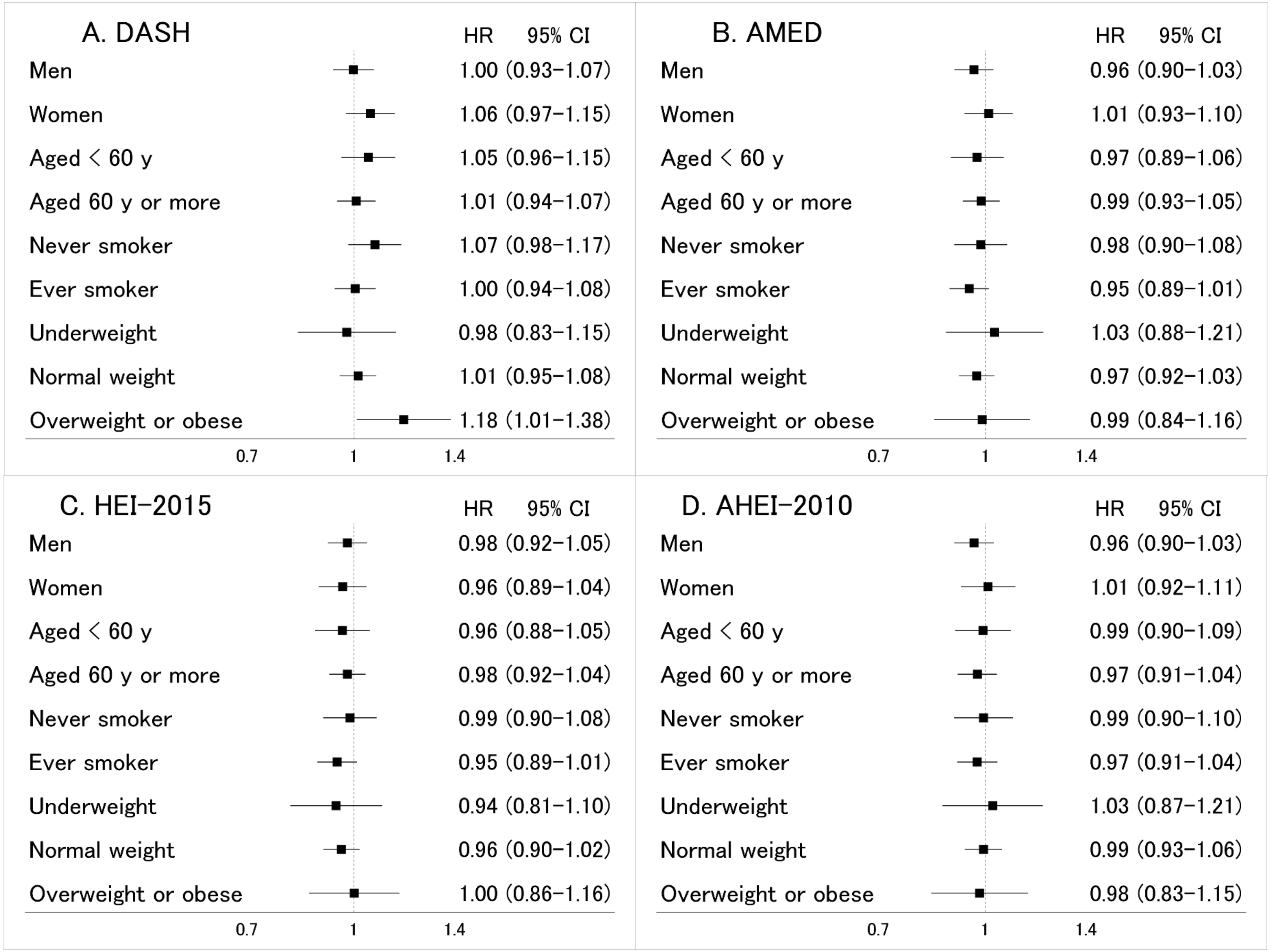
A. DASH
HR 95% CI
Men 1.00 (0.93–1.07)
Women 1.06 (0.97–1.15)
Aged < 60 y 1.05 (0.96–1.15)
Aged 60 y or more 1.01 (0.94–1.07)
Never smoker 1.07 (0.98–1.17)
Ever smoker 1.00 (0.94–1.08)
Underweight 0.98 (0.83–1.15)
Normal weight 1.01 (0.95–1.08)
Overweight or obese 1.18 (1.01–1.38)
0.7 1 1.4
B. AMED
HR 95% CI
Men 0.96 (0.90–1.03)
Women 1.01 (0.93–1.10)
Aged < 60 y 0.97 (0.89–1.06)
Aged 60 y or more 0.99 (0.93–1.05)
Never smoker 0.98 (0.90–1.08)
Ever smoker 0.95 (0.89–1.01)
Underweight 1.03 (0.88–1.21)
Normal weight 0.97 (0.92–1.03)
Overweight or obese 0.99 (0.84–1.16)
0.7 1 1.4
C. HEI-2015
HR 95% CI
Men 0.98 (0.92–1.05)
Women 0.96 (0.89–1.04)
Aged < 60 y 0.96 (0.88–1.05)
Aged 60 y or more 0.98 (0.92–1.04)
Never smoker 0.99 (0.90–1.08)
Ever smoker 0.95 (0.89–1.01)
Underweight 0.94 (0.81–1.10)
Normal weight 0.96 (0.90–1.02)
Overweight or obese 1.00 (0.86–1.16)
0.7 1 1.4
D. AHEI-2010
HR 95% CI
Men 0.96 (0.90–1.03)
Women 1.01 (0.92–1.11)
Aged < 60 y 0.99 (0.90–1.09)
Aged 60 y or more 0.97 (0.91–1.04)
Never smoker 0.99 (0.90–1.10)
Ever smoker 0.97 (0.91–1.04)
Underweight 1.03 (0.87–1.21)
Normal weight 0.99 (0.93–1.06)
Overweight or obese 0.98 (0.83–1.15)
0.7 1 1.4

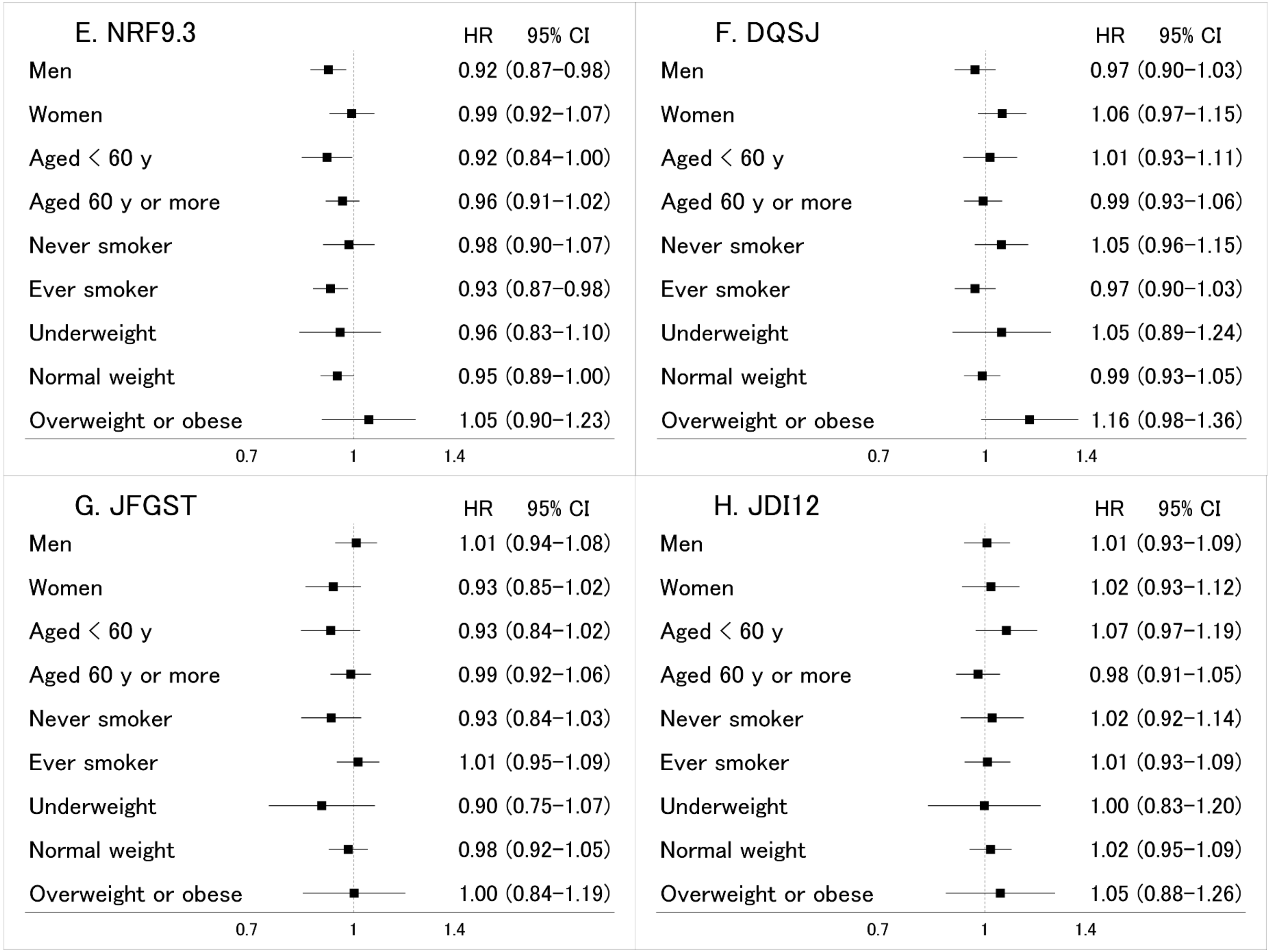
E. NRF9.3
HR 95% CI
Men 0.92 (0.87–0.98)
Women 0.99 (0.92–1.07)
Aged < 60 y 0.92 (0.84–1.00)
Aged 60 y or more 0.96 (0.91–1.02)
Never smoker 0.98 (0.90–1.07)
Ever smoker 0.93 (0.87–0.98)
Underweight 0.96 (0.83–1.10)
Normal weight 0.95 (0.89–1.00)
Overweight or obese 1.05 (0.90–1.23)
0.7
1
1.4
F. DQSJ
HR 95% CI
Men 0.97 (0.90–1.03)
Women 1.06 (0.97–1.15)
Aged < 60 y 1.01 (0.93–1.11)
Aged 60 y or more 0.99 (0.93–1.06)
Never smoker 1.05 (0.96–1.15)
Ever smoker 0.97 (0.90–1.03)
Underweight 1.05 (0.89–1.24)
Normal weight 0.99 (0.93–1.05)
Overweight or obese 1.16 (0.98–1.36)
0.7
1
1.4
G. JFGST
HR 95% CI
Men 1.01 (0.94–1.08)
Women 0.93 (0.85–1.02)
Aged < 60 y 0.93 (0.84–1.02)
Aged 60 y or more 0.99 (0.92–1.06)
Never smoker 0.93 (0.84–1.03)
Ever smoker 1.01 (0.95–1.09)
Underweight 0.90 (0.75–1.07)
Normal weight 0.98 (0.92–1.05)
Overweight or obese 1.00 (0.84–1.19)
0.7
1
1.4
H. JDI12
HR 95% CI
Men 1.01 (0.93–1.09)
Women 1.02 (0.93–1.12)
Aged < 60 y 1.07 (0.97–1.19)
Aged 60 y or more 0.98 (0.91–1.05)
Never smoker 1.02 (0.92–1.14)
Ever smoker 1.01 (0.93–1.09)
Underweight 1.00 (0.83–1.20)
Normal weight 1.02 (0.95–1.09)
Overweight or obese 1.05 (0.88–1.26)
0.7
1
1.4

Supplemental Figure 5 Hazard ratios (HR) on cancer mortality per 1 SD increment of each diet quality indices in the Takayama study, stratified by basic characteristics (sex, age, smoking status, and weight status) [a]

DASH, Dietary Approaches to Stop Hypertension; AMED, Alternate Mediterranean Diet; HEI-2015, Healthy Eating Index-2015; AHEI-2010, Alternate Healthy Eating Index-2010; NRF9.3, Nutrient Rich Food Score 9.3; DQSJ, Diet Quality Score for Japanese; JFGST, Japanese Food Guide Spinning Top; JDI12, 12-item Japanese Diet Index.

[a] The horizontal axis for HR is scaled logarithmically. Cox proportional hazards models to estimate HRs and 95% CIs were adjusted for age, sex, total energy intake (continuous), body mass index (continuous, as quadratic term), physical activity (continuous), smoking (pack year, continuous), education (less than 12 years, 12–14 years, 15 years or more), marital status, sleep duration (continuous, as quadratic term), history of hypertension (yes or no), history of diabetes (yes or no), multivitamin use (yes or no), menopause status (yes or no, only for women). Participant numbers and cases were 13,355 and 974 for men, 15,724 and 646 for women, 19,128 and 518 for participants aged less than 60 y, 9,951 and 1,102 for participants aged 60 y or more, 13,802 and 528 for never smokers, 13,257 and 959 for ever smokers, 3,895 and 287 for underweight, 21,101 and 1,162 for normal weight, and 4,083 and 171 for overweight. No significant interactions on cancer mortality were observed between the diet quality indices and basic characteristics.

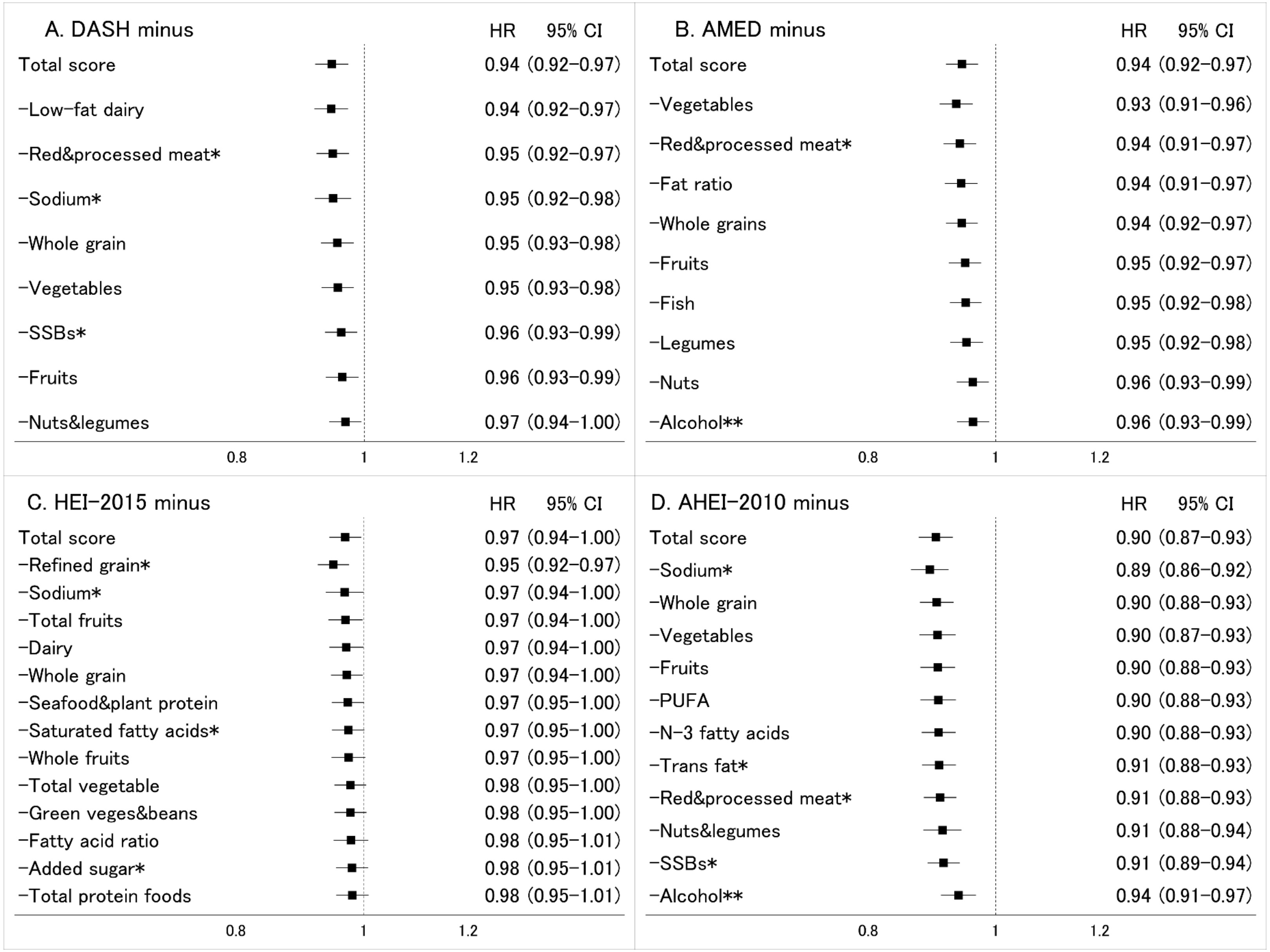
A. DASH minus
HR 95% CI
Total score 0.94 (0.92–0.97)
–Low–fat dairy 0.94 (0.92–0.97)
–Red&processed meat* 0.95 (0.92–0.97)
–Sodium* 0.95 (0.92–0.98)
–Whole grain 0.95 (0.93–0.98)
–Vegetables 0.95 (0.93–0.98)
–SSBs* 0.96 (0.93–0.99)
–Fruits 0.96 (0.93–0.99)
–Nuts&legumes 0.97 (0.94–1.00)
0.8
1
1.2
B. AMED minus
HR 95% CI
Total score 0.94 (0.92–0.97)
–Vegetables 0.93 (0.91–0.96)
–Red&processed meat* 0.94 (0.91–0.97)
–Fat ratio 0.94 (0.91–0.97)
–Whole grains 0.94 (0.92–0.97)
–Fruits 0.95 (0.92–0.97)
–Fish 0.95 (0.92–0.98)
–Legumes 0.95 (0.92–0.98)
–Nuts 0.96 (0.93–0.99)
–Alcohol** 0.96 (0.93–0.99)
0.8
1
1.2
C. HEI–2015 minus
HR 95% CI
Total score 0.97 (0.94–1.00)
–Refined grain* 0.95 (0.92–0.97)
–Sodium* 0.97 (0.94–1.00)
–Total fruits 0.97 (0.94–1.00)
–Dairy 0.97 (0.94–1.00)
–Whole grain 0.97 (0.94–1.00)
–Seafood&plant protein 0.97 (0.95–1.00)
–Saturated fatty acids* 0.97 (0.95–1.00)
–Whole fruits 0.97 (0.95–1.00)
–Total vegetable 0.98 (0.95–1.00)
–Green veges&beans 0.98 (0.95–1.00)
–Fatty acid ratio 0.98 (0.95–1.01)
–Added sugar* 0.98 (0.95–1.01)
–Total protein foods 0.98 (0.95–1.01)
0.8
1
1.2
D. AHEI–2010 minus
HR 95% CI
Total score 0.90 (0.87–0.93)
–Sodium* 0.89 (0.86–0.92)
–Whole grain 0.90 (0.88–0.93)
–Vegetables 0.90 (0.87–0.93)
–Fruits 0.90 (0.88–0.93)
–PUFA 0.90 (0.88–0.93)
–N–3 fatty acids 0.90 (0.88–0.93)
–Trans fat* 0.91 (0.88–0.93)
–Red&processed meat* 0.91 (0.88–0.93)
–Nuts&legumes 0.91 (0.88–0.94)
–SSBs* 0.91 (0.89–0.94)
–Alcohol** 0.94 (0.91–0.97)
0.8
1
1.2

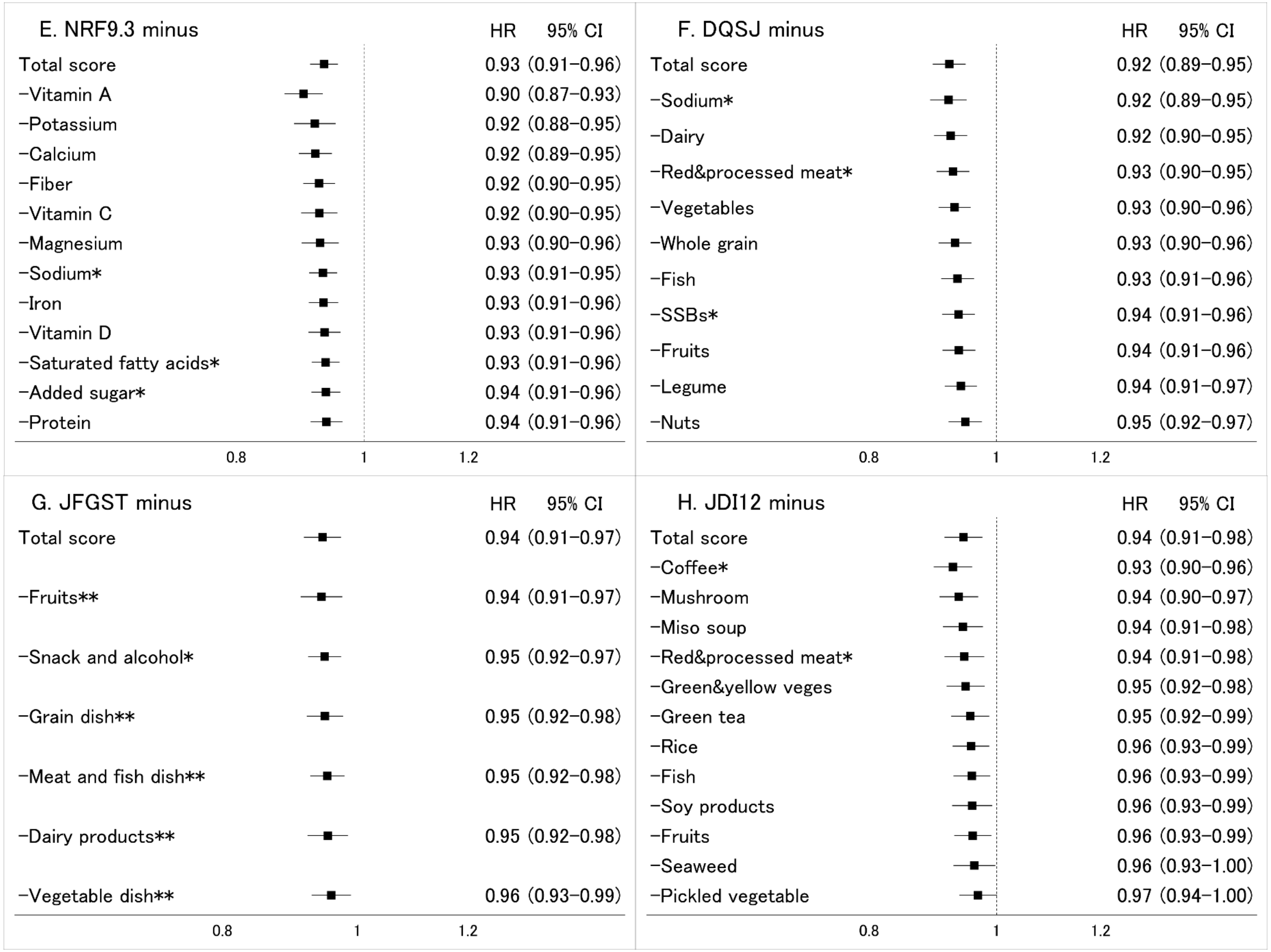
E. NRF9.3 minus
HR 95% CI
Total score 0.93 (0.91–0.96)
–Vitamin A 0.90 (0.87–0.93)
–Potassium 0.92 (0.88–0.95)
–Calcium 0.92 (0.89–0.95)
–Fiber 0.92 (0.90–0.95)
–Vitamin C 0.92 (0.90–0.95)
–Magnesium 0.93 (0.90–0.96)
–Sodium* 0.93 (0.91–0.95)
–Iron 0.93 (0.91–0.96)
–Vitamin D 0.93 (0.91–0.96)
–Saturated fatty acids* 0.93 (0.91–0.96)
–Added sugar* 0.94 (0.91–0.96)
–Protein 0.94 (0.91–0.96)
0.8
1
1.2
F. DQSJ minus
HR 95% CI
Total score 0.92 (0.89–0.95)
–Sodium* 0.92 (0.89–0.95)
–Dairy 0.92 (0.90–0.95)
–Red&processed meat* 0.93 (0.90–0.95)
–Vegetables 0.93 (0.90–0.96)
–Whole grain 0.93 (0.90–0.96)
–Fish 0.93 (0.91–0.96)
–SSBs* 0.94 (0.91–0.96)
–Fruits 0.94 (0.91–0.96)
–Legume 0.94 (0.91–0.97)
–Nuts 0.95 (0.92–0.97)
0.8
1
1.2
G. JFGST minus
HR 95% CI
Total score 0.94 (0.91–0.97)
–Fruits** 0.94 (0.91–0.97)
–Snack and alcohol* 0.95 (0.92–0.97)
–Grain dish** 0.95 (0.92–0.98)
–Meat and fish dish** 0.95 (0.92–0.98)
–Dairy products** 0.95 (0.92–0.98)
–Vegetable dish** 0.96 (0.93–0.99)
0.8
1
1.2
H. JDI12 minus
HR 95% CI
Total score 0.94 (0.91–0.98)
–Coffee* 0.93 (0.90–0.96)
–Mushroom 0.94 (0.90–0.97)
–Miso soup 0.94 (0.91–0.98)
–Red&processed meat* 0.94 (0.91–0.98)
–Green&yellow veges 0.95 (0.92–0.98)
–Green tea 0.95 (0.92–0.99)
–Rice 0.96 (0.93–0.99)
–Fish 0.96 (0.93–0.99)
–Soy products 0.96 (0.93–0.99)
–Fruits 0.96 (0.93–0.99)
–Seaweed 0.96 (0.93–1.00)
–Pickled vegetable 0.97 (0.94–1.00)
0.8
1
1.2

Supplement Figure 6 Hazard ratios (HR) on all-cause mortality per 1 SD increment of the total minus each component score of the selected three diet quality indices in the Takayama study [a]

DASH, Dietary Approaches to Stop Hypertension; AMED, Alternate Mediterranean Diet; HEI-2015, Healthy Eating Index-2015; AHEI-2010, Alternate Healthy Eating Index-2010; NRF9.3, Nutrient Rich Food Score 9.3; DQSJ, Diet Quality Score for Japanese; JFGST, Japanese Food Guide Spinning Top; JDI12, 12-item Japanese Diet Index; SFA, saturated fatty acids; SSBs, sugar-sweetened beverages.

[a] The horizontal axis for HR is scaled logarithmically. Cox proportional hazards models to estimate HRs and 95% CIs were adjusted for age, sex, total energy intake (continuous), body mass index (continuous, as quadratic term), physical activity (continuous), smoking (pack year, continuous), education (less than 12 years, 12–14 years, 15 years or more), marital status, sleep duration (continuous, as quadratic term), history of hypertension (yes or no), history of diabetes (yes or no), multivitamin use (yes or no), menopause status (yes or no, only for women) and each removed component score.

N of participants and deaths were 29,079 and 5,339.

[*] Lower intake receives a higher component score

[**] Intake within the pre-defined range receives a higher component score

References

1. Oono F, Murakami K, Fujiwara A, et al. Development of a Diet Quality Score for Japanese and Comparison With Existing Diet Quality Scores Regarding Inadequacy of Nutrient Intake. J Nutr. 2023;153(3):798-810. doi:10.1016/j.tjnut.2022.11.022

2. Fung TT, Chiuve SE, McCullough ML, Rexrode KM, Logroscino G, Hu FB. Adherence to a DASH-style diet and risk of coronary heart disease and stroke in women. Arch Intern Med. 2008;168(7):713-20. doi:10.1001/archinte.168.7.713

3. Abdelhamid A, Jennings A, Hayhoe RPG, Awuzudike VE, Welch AA. High variability of food and nutrient intake exists across the Mediterranean Dietary Pattern-A systematic review. Food Sci Nutr. 2020;8(9):4907-18. doi:10.1002/fsn3.1784

4. Hutchins-Wiese HL, Bales CW, Porter Starr KN. Mediterranean diet scoring systems: understanding the evolution and applications for Mediterranean and non-Mediterranean countries. Br J Nutr. 2022;128(7):1371-92. doi:10.1017/S0007114521002476

5. Soltani S, Arablou T, Jayedi A, Salehi-Abargouei A. Adherence to the dietary approaches to stop hypertension (DASH) diet in relation to all-cause and cause-specific mortality: a systematic review and dose-response meta-analysis of prospective cohort studies. Nutr J. 2020;19(1):37. doi:10.1186/s12937-020-00554-8

6. Fung TT, McCullough ML, Newby PK, et al. Diet-quality scores and plasma concentrations of markers of inflammation and endothelial dysfunction. Am J Clin Nutr. 2005;82(1):163-73. doi:10.1093/ajcn.82.1.163

7. Krebs-Smith SM, Pannucci TE, Subar AF, et al. Update of the Healthy Eating Index: HEI-2015. J Acad Nutr Diet. 2018;118(9):1591-602. doi:10.1016/j.jand.2018.05.021

8. Murakami K, Livingstone MBE, Fujiwara A, Sasaki S. Reproducibility and Relative Validity of the Healthy Eating Index-2015 and Nutrient-Rich Food Index 9.3 Estimated by Comprehensive and Brief Diet History Questionnaires in Japanese Adults. Nutrients. 2019;11(10). doi:10.3390/nu11102540

9. Bowman SA, Clemens JC, Thoerig RC, Friday JE, Shimizu M, Moshfegh AJ. Food Patterns Equivalents Database 2011-12 : Methodology and User Guide. Food Surveys Research Group, Beltsville Human Nutrition Research Center, Agricultural Research Service, U.S. Department of Agriculture. 2014. http://www.ars.usda.gov/ba/bhnrc/fsrg%0D.

10. Chiuve SE, Fung TT, Rimm EB, et al. Alternative dietary indices both strongly predict risk of chronic disease. J Nutr. 2012;142(6):1009-18. doi:10.3945/jn.111.157222

11. Aune D, Giovannucci E, Boffetta P, et al. Fruit and vegetable intake and the risk of cardiovascular disease, total cancer and all-cause mortality-a systematic review and dose-response meta-analysis of prospective studies. Int J Epidemiol. 2017;46(3):1029-56. doi:10.1093/ije/dyw319

12. U.S. Department of Agriculture and U.S. Department of Health and Human Services. Dietary Guidelines for Americans 2015-2020. 8th Edition 2015 [Available from: https://health.gov/our-work/food-nutrition/previous-dietary-guidelines/2015. accessed 9th Sep

2023
13. Fulgoni VL, 3rd, Keast DR, Drewnowski A. Development and validation of the nutrient-rich foods index: a tool to measure nutritional quality of foods. J Nutr. 2009;139(8):1549-54. doi:10.3945/jn.108.101360
14. Ministry of Health LaWJ. Dietary Reference Intakes for Japanese, 2020. Ministry of Health, Labour and Welfare Japan. 2019. https://www.mhlw.go.jp/stf/newpage_08517.html. Accessed 28th Mar 2022.
15. World Health Organization. Guideline: Sugars intake for adults and children. Geneva: World Health Organization; 2015. World Health Organization. 2015. http://apps.who.int/iris/bitstream/10665/149782/1/9789241549028_eng.pdf?ua=1. Accessed 1st Jul 2020.
16. Oba S, Nagata C, Nakamura K, et al. Diet based on the Japanese Food Guide Spinning Top and subsequent mortality among men and women in a general Japanese population. J Am Diet Assoc. 2009;109(9):1540-7. doi:10.1016/j.jada.2009.06.367
17. Shimizu H, Ohwaki A, Kurisu Y, et al. Validity and reproducibility of a quantitative food frequency questionnaire for a cohort study in Japan. Jpn J Clin Oncol. 1999;29(1):38-44. doi:10.1093/jjco/29.1.38
18. Subar AF, Freedman LS, Tooze JA, et al. Addressing Current Criticism Regarding the Value of Self-Report Dietary Data. J Nutr. 2015;145(12):2639-45. doi:10.3945/jn.115.219634
19. Zhang S, Otsuka R, Tomata Y, et al. A cross-sectional study of the associations between the traditional Japanese diet and nutrient intakes: the NILS-LSA project. Nutr J. 2019;18(1):43. doi:10.1186/s12937-019-0468-9
20. Saji N, Tsuduki T, Murotani K, et al. Relationship between the Japanese-style diet, gut microbiota, and dementia: A cross-sectional study. Nutrition. 2022;94:111524. doi:10.1016/j.nut.2021.111524
